# Gene selection algorithms for microarray data based on least squares support vector machine

**DOI:** 10.1186/1471-2105-7-95

**Published:** 2006-02-27

**Authors:** E Ke Tang, PN Suganthan, Xin Yao

**Affiliations:** 1 School of Electrical & Electronic Engineering, Nanyang Technological University, Singapore; 2 School of Computer Science, University of Birmingham, Birmingham B15 2TT, UK

## Abstract

**Background:**

In discriminant analysis of microarray data, usually a small number of samples are expressed by a large number of genes. It is not only difficult but also unnecessary to conduct the discriminant analysis with all the genes. Hence, gene selection is usually performed to select important genes.

**Results:**

A gene selection method searches for an optimal or near optimal subset of genes with respect to a given evaluation criterion. In this paper, we propose a new evaluation criterion, named the leave-one-out calculation (LOOC, A list of abbreviations appears just above the list of references) measure. A gene selection method, named leave-one-out calculation sequential forward selection (LOOCSFS) algorithm, is then presented by combining the LOOC measure with the sequential forward selection scheme. Further, a novel gene selection algorithm, the gradient-based leave-one-out gene selection (GLGS) algorithm, is also proposed. Both of the gene selection algorithms originate from an efficient and exact calculation of the leave-one-out cross-validation error of the least squares support vector machine (LS-SVM). The proposed approaches are applied to two microarray datasets and compared to other well-known gene selection methods using codes available from the second author.

**Conclusion:**

The proposed gene selection approaches can provide gene subsets leading to more accurate classification results, while their computational complexity is comparable to the existing methods. The GLGS algorithm can also better scale to datasets with a very large number of genes.

## Background

Recently, discriminant analysis of microarray data has been widely used to assist diagnosis [1-3]. Given some microarray data characterized by a large number of genes' expressions, a typical discriminant analysis constructs a classifier based on the given data to distinguish between different disease types. In practice, a gene selection procedure to select the most informative genes from the whole gene set is usually employed. There are several reasons for performing gene selection. First, the cost of clinical diagnosis can be reduced with gene selection since it is much cheaper to focus on only the expressions of a few genes for diagnosis instead of the whole gene set. Second, many of the genes in the whole gene set are redundant. Although the training error of a classifier on the given data will decrease as more and more genes are included, the generalization error when classifying new data eventually will increase. A preceding gene selection procedure can remove the redundant genes, reduce storage requirement and computational complexity of the following discriminant analysis, and possibly reduce the generalization error. Finally, gene selection can provide a more compact gene set, which can help understand the functions of particular genes and plan the diagnosis process.

From the perspective of pattern recognition, the gene selection problem is a special case of the feature selection problem. Given a set of training data represented by a set of features, a typical feature selection method aims to select a feature subset leading to a low generalization error (i.e. low error in future classification). It searches for an optimal or near optimal subset of features with respect to a given criterion, and thus consists of two basic components: an evaluation criterion and a search scheme. Feature selection methods can generally be categorized into three major groups: marginal filters, wrappers and embedded methods [4]. Marginal filter approaches are usually mentioned as individual feature ranking methods. They evaluate a feature based on its marginal contribution to the class discrimination without considering its interactions with other features. The selection procedure is independent of the classification procedure because a classifier is not built when evaluating a feature. Some comparative studies on the criteria employed in a marginal filter method can be found in [5]. In a wrapper method, usually a classifier is built and employed as the evaluation criterion. One such example is to use the training or cross-validation error of the classifier on the training data as the evaluation criterion. Because the finally selected feature subset has the highest value on the criterion, the feature selection procedure is closely related to the decision mechanism of the classifier and therefore the wrapper methods are expected to generate better feature subsets for classification than the marginal filter methods. If the criterion is derived from the intrinsic properties of a classifier, the corresponding feature selection method will be categorized as an embedded approach [6]. For example, in the SVM Recursive Feature Elimination (SVM-RFE) algorithm, a support vector machine is trained first. then the features corresponding to the smallest weights in the vector normal to the optimal hyperplane is sequentially eliminated [7]. Nevertheless, wrapper and embedded methods are often closely related to each other.

Because the marginal filter methods evaluate features separately and there is no other search scheme for them, we mainly discuss the search schemes for the wrapper and embedded methods. In a wrapper or embedded feature selection algorithm, if the whole feature subset space is explicitly (such as using an exhaustive search) or implicitly searched (such as using the branch-and-bound scheme [8]), it is guaranteed to discover the optimal feature subset with respect to the evaluation criterion. However, an exhaustive search or the branch-and-bound scheme is computationally prohibitive except for small problems. Therefore, one usually searches a part of the whole feature subset space, which is more practicable but provide no optimality guarantees [9]. The sequential forward selection, sequential floating forward selection, sequential backward elimination, sequential floating backward elimination and so on [10] belong to this class. The sequential forward scheme starts from an empty set, and sequentially includes a new feature into the feature subset so that the largest improvement on the evaluation criterion can be achieved. Once a feature is selected, it will not be removed from the subset. Differently, the sequential floating forward selection scheme contains two steps. First, the feature leading to the largest improvement is included, then the scheme backtracks the search path and removes some previously selected features if improvement can be achieved by doing so. Sequential backward elimination scheme sequentially remove features from the whole feature set until an optimal feature subset is remained. And the sequential floating backward elimination allows including previously removed features to the current feature subset. Hence, the floating schemes cover a larger portion of all the possible feature subsets, while it is more time consuming [10]. Recently, genetic algorithms (GA) have also been employed as the search schemes [11-13]. Compared with the traditional search schemes, GAs provide a more flexible search procedure, the feature subset space is searched in parallel and multiple feature subsets instead of a single subset are evaluated simultaneously to avoid being trapped in a local optimum. GAs are generally even more time consuming than the floating schemes, although it can cover more feature subsets.

In the context of microarray data analysis, many of the methodologies discussed above have been used. In addition to those marginal filter methods using t-statistics, Fisher's ratio and information gain, different evaluation criteria were proposed for wrapper and embedded methods, such as the SVM-based criteria [14] and the LS bound measure, which is based on a lower bound of the leave-one-out cross-validation error of the least squares support vector machine (LS-SVM) [15]. They can be combined with any kinds of search scheme. Several GA-based algorithms are also available in the literature of gene selection [12,13,16]. Two issues should be considered when assessing these gene selection methods: the generalization error that can be achieved on the selected gene subset and the time requirement of the selection procedure. Specifically, a good feature selection method should contain following characteristics: The evaluation criterion can guarantee low generalization error, computational cost for a single evaluation is low, the search scheme requires a small number of evaluations while can still search a large portion of the whole feature subset space in order to include the optimal ones. Therefore, among all the methods discussed above, the marginal filter methods are the most efficient, but the selected feature subsets are usually sub-optimal. The wrapper/embedded methods using an exhaustive search are the most time consuming, but optimality can be guaranteed. All the other methods lie between these two cases, providing a trade-off between optimality and computation cost. The wrapper methods using sequential selection schemes are more efficient than the wrapper methods using GA based algorithms, but are more likely to select a sub-optimal gene subset. Furthermore, in addition to finding an optimal gene subset for classification, identifying important genes is another goal of gene selection. Identifying important genes is essentially different from finding a single optimal gene subset. For the microarray data, a classifier may be able to achieve the lowest generalization error on many different gene subsets, and all of them consist of important genes. Knowing these different gene subsets can help gain more insight into the functions of genes.

In the present study, we first propose an evaluation criterion called leave-one-out calculation (LOOC) measure for gene selection. The LOOC measure is derived from an exact and efficient calculation of the leave-one-out cross-validation error (LOOE) of LS-SVM. By combining the LOOC measure with the sequential forward selection scheme, we proposed the leave-one-out calculation sequential forward selection (LOOCSFS) gene selection algorithm. Moreover, we also present a novel gene selection algorithm, named gradient-based leave-one-out gene selection (GLGS) algorithm. Employing none of the traditional search schemes, it combines a variant of the LOOC measure with the gradient descent optimization and the principal component analysis (PCA). Performance of the proposed methods is evaluated experimentally on two microarray datasets.

## Results

### Datasets

In this section, we present performance of the proposed gene selection algorithms, i.e. the LOOCSFS and the GLGS algorithms on two public domain datasets.

#### Hepatocellular carcinoma dataset

This dataset comprises information of 60 patients with hepatocellular, with oligonucleotide microarrays representing 7129 gene expression levels [2].

#### Glioma dataset

All the 50 samples of the Glioma dataset [3] are expressed by 12625 genes. Twenty eight of the samples are glioblastomas and the other 22 are anaplastic oligodendrogliomas.

### Experimental setup

We acquire the two datasets directly from [17]. We further standardize the data to zero mean and unit standard deviation for each gene. The experiments and results are based on the pre-processed data and are implemented in the Matlab environment on a computer with 3 GHz P4 CPU and 1024 MB RAM.

There are two objectives for the experiments. One is to evaluate the performance of LOOCSFS and GLGS algorithms, and compare them with other gene selection algorithms. The other goal of the experiments is to identify important genes of the two datasets.

For the first objective, we compare our leave-one-out calculation sequential forward selection (LOOCSFS) and gradient-based leave-one-out gene selection (GLGS) algorithms with other five gene selection algorithms. First, although it usually selects a sub-optimal gene subset for classification, a marginal filter method using Fisher's ratio is employed to provide a baseline for the comparison on generalization errors. Fisher's ratio is a criterion that evaluates how well a single gene is correlated with the separation between classes. For every gene the Fisher's ratio is defined as f(μ1−μ2)2σ12+σ22
 MathType@MTEF@5@5@+=feaafiart1ev1aaatCvAUfKttLearuWrP9MDH5MBPbIqV92AaeXatLxBI9gBaebbnrfifHhDYfgasaacH8akY=wiFfYdH8Gipec8Eeeu0xXdbba9frFj0=OqFfea0dXdd9vqai=hGuQ8kuc9pgc9s8qqaq=dirpe0xb9q8qiLsFr0=vr0=vr0dc8meaabaqaciaacaGaaeqabaqabeGadaaakeaacqWGMbGzdaWcaaqaaiabcIcaOiabeY7aTnaaBaaaleaacqaIXaqmaeqaaOGaeyOeI0IaeqiVd02aaSbaaSqaaiabikdaYaqabaGccqGGPaqkdaahaaWcbeqaaiabikdaYaaaaOqaaiabeo8aZnaaDaaaleaacqaIXaqmaeaacqaIYaGmaaGccqGHRaWkcqaHdpWCdaqhaaWcbaGaeGOmaidabaGaeGOmaidaaaaaaaa@4025@, where *μ*_1_, *μ*_2_, σ_1_, σ_2 _denote the means and standard deviations of two classes. Further, our methods are compared with the Mahalanobis class separability measure (MAHSFS) [8] and the LS bound measure combined with sequential forward selection scheme (LSSFS) [15], SVM-RFE [7], the LS bound measure with sequential floating forward selection scheme (LSSFFS) [15]. The comparisons are conducted based on the generalization error achieved on the selected gene subset and the time requirement of the selection procedure. In two previous works, Ambroise and McLachlan and Simon *et al*. demonstrated that cross-validation or bootstrap samples should be kept external to a gene selection algorithm [18,19]. Ambroise and McLachlan [18] also assessed several techniques for estimating the generalization error. They showed that the external 10-fold cross-validation error and the external B.632+ error are the two most unbiased estimators of the generalization error. Since cross-validation is claimed to have a relatively higher variance for small sample size problems [20], the external B.632+ error appears to be the best choice. Hence we use the external B.632+ technique [21] to compare the generalization error achieved on the selected gene subsets. The B.632+ technique employs the bagging [22] procedure to generate different training and testing sets (which are called bootstrap samples [21]) from the original data. The algorithms are applied to the bootstrap samples as well as the original data. Specifically, we employ 200 replicates of balanced bootstrap samples to reduce variance of the B.632+ error, i.e. each sample in the original dataset is restricted to appear exactly 200 times in total in all the 200 balanced bootstrap samples. A standard SVM is employed as the final classifier for all seven gene selection methods. All the compared algorithms will terminate if a predefined number of genes are selected. We set this number as 100. Furthermore, we conduct another experiment to study the computational complexity and scalability of the seven gene selection algorithms. The required computational time of the algorithms are studied with respect to the number of genes to be selected (*t*) and the size of the whole gene set (*d*). This experiment is conducted on the Hepatocellular Carcinoma dataset, but similar scenario can be easily shown on the Glioma dataset.

The second objective of identifying important genes is essentially different from selecting a single gene subset for classification. Some researchers claimed that the gene selection procedure should not be applied only once on the original data, but should be run repeatedly on different subsets of the training data [13,23-25]. Since we apply our algorithms to 200 bootstrap samples for the first objective, we can actually achieve 200 different gene subsets for these bootstrap samples. By looking at the frequency of genes appearing in all the 200 gene subsets, we can find some insights on the genes that are important for classification. Therefore, for both LOOCSFS and GLGS algorithms, we identify the top 20 genes that are most frequently selected based on the 200 bootstrap samples.

### Results

**Figure 1:**
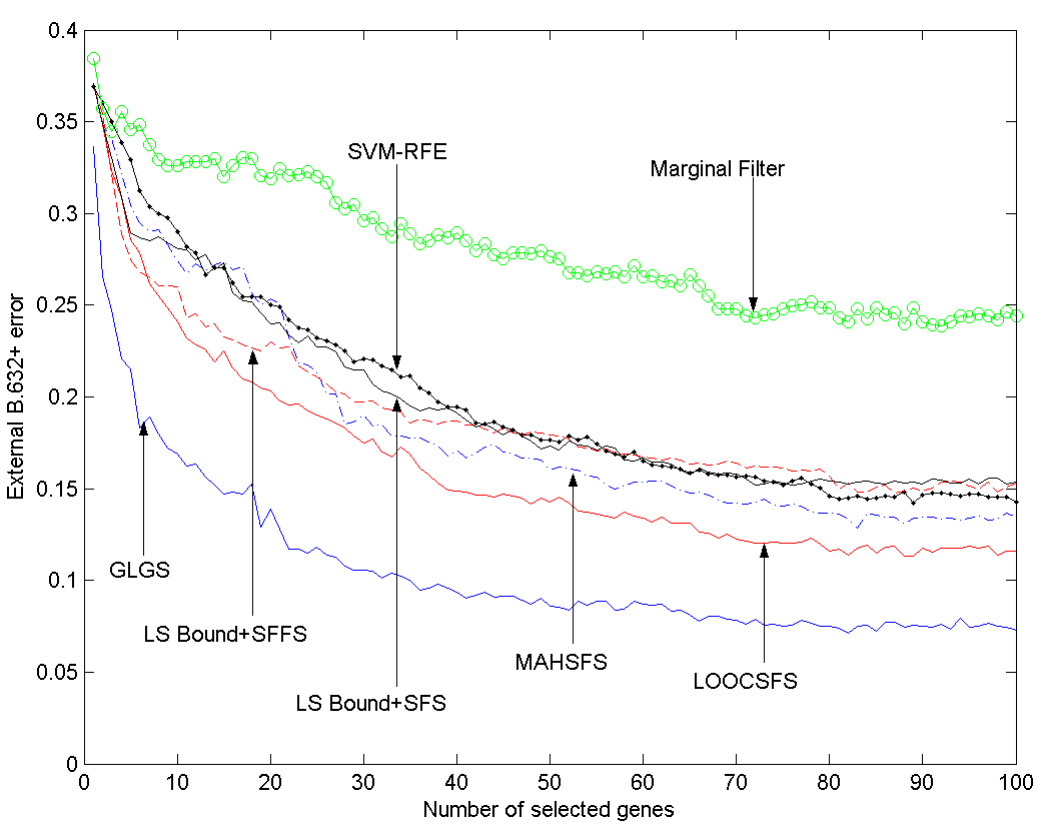
The external B.632+ error for Hepatocellular Carcinoma dataset, shown vs the number of selected genes.

**Figure 2:**
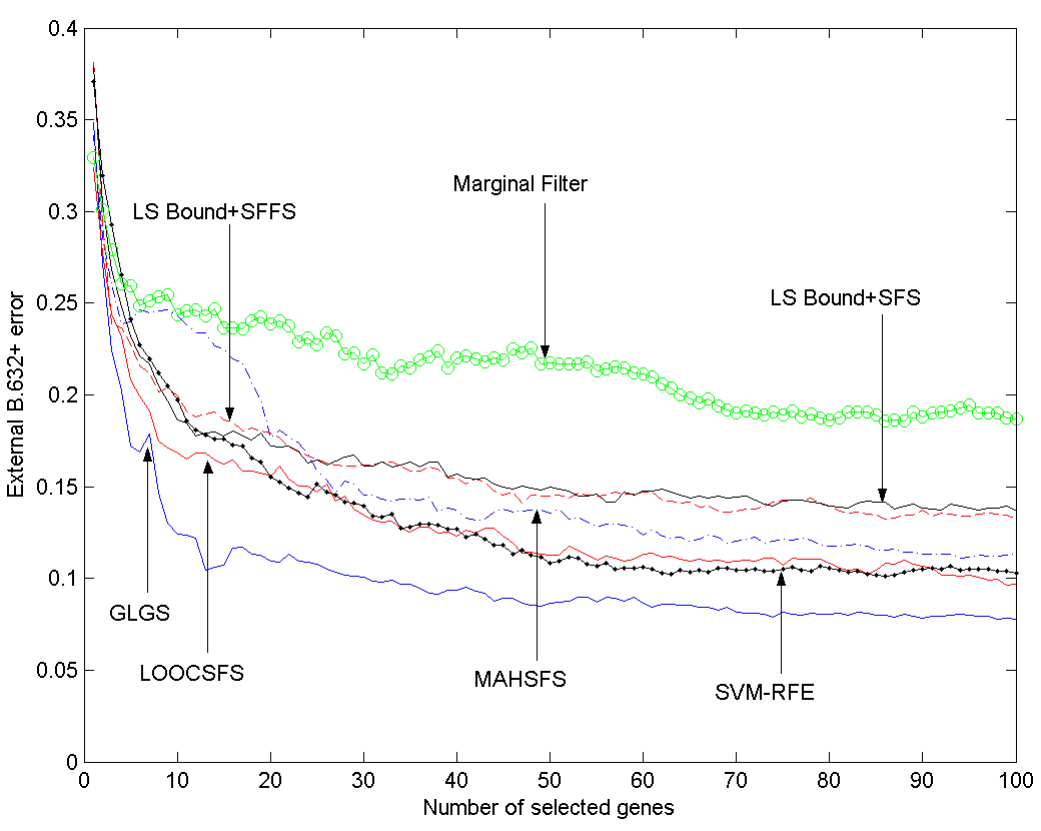
The external B.632+ error for Glioma dataset, shown vs the number of selected genes.

Figures 1 and 2 present the external B.632+ errors achieved on the genes selected by the seven gene selection algorithms. It can be observed that the GLGS algorithm generally achieves the lowest external B.632+ error among the compared methods on both datasets. The LOOCSFS algorithm does not perform as well as the GLGS algorithm. As shown in the figures, LOOCSFS is consistently superior to the marginal filter method, LSSFS. It also outperforms the MAHSFS and SVM-RFE on the Hepatocellular Carcinoma datasets, and the results are mixed on the Glioma dataset. Furthermore, although a gene selection algorithm employing the sequential forward selection scheme is expected to be inferior to the methods employing the sequential floating forward selection scheme (because the sequential forward selection scheme searches a smaller portion of the feature subset space than the sequential floating forward selection scheme), it also outperforms the LSSFFS on both datasets.

**Figure 3:**
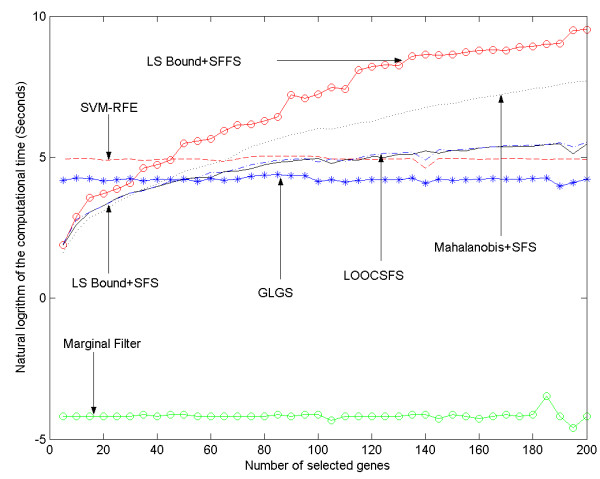
The computational time of seven gene selection algorithms on Hepatocellular Carcinoma dataset, shown vs the number of selected genes.

**Figure 4:**
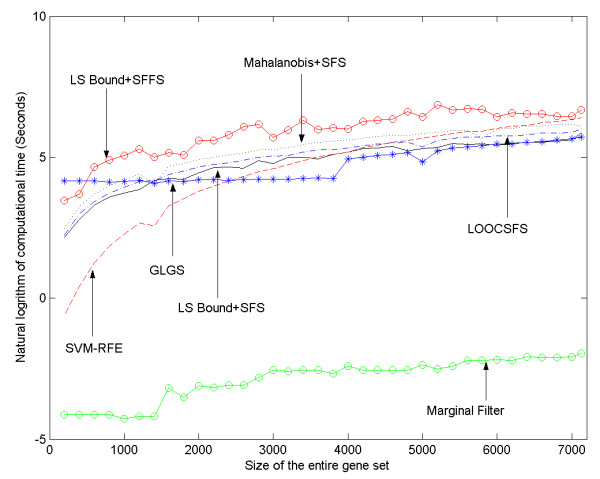
The computational time of seven gene selection algorithms on Hepatocellular Carcinoma dataset, shown vs the size of the gene set.

From the perspective of computational complexity, the scalability of a gene selection algorithm should also be considered when evaluating it. The required computational time of the algorithms are plotted with respect to the number of genes to be selected (*t*) and the size of the whole gene set (*d*) in Figures 3 and 4. As shown in the two figures, the marginal filter method is always the most efficient one among all the approaches. The computational costs of LSSFS, MAHSFS, LOOCSFS and LSSFFS all increase significantly when *d *or *t *increases. In Fig. 3, LSSFFS is the most time consuming since the sequentially floating forward selection scheme is employed. MAHSFS also requires expensive computation. The LSSFS and the LOOCSFS can be calculated more efficiently than LSSFFS and MAHSFS. The LOOCSFS requires slightly more time than the LSSFS. The computational time of SVM-RFE and GLGS do not change significantly with *t*, and SVM-RFE is more time consuming than GLGS. In Figure 4, computational costs of all methods except GLGS increase significantly with *d*, with the LSSFFS being the most time consuming and the other four are comparable. Hence the GLGS algorithm can better scale to microarray data with large number of genes as well as the problems that require selecting a large number of genes from the original gene set.

#### Hepatocellular carcinoma dataset

The 20 genes most frequently selected by LOOCSFS and GLGS are listed in Table 1 and 2 respectively. Some comments about the selected genes are worthy of mention. Genes M59465, X75042, Y10032, L08895, AB000409, L11695, X15341 and L76927 are frequently selected by both algorithms. Among them, M59465, X75042, Y10032 and L08895 are also used to construct an SVM classifier in the original work [2]. The Y10032 and M59465 are claimed as greatly downregulated in hepatocellular carcinoma with venous invasion and the levels of Y10032 transcript are altered in hepatoma cells in response to osmotic changes or cell volume changes [2]. In addition, LOOCSFS also frequently select gene X00274, which is downregulated in hepatocellular carcinomas with early intrahepatic recurrence and this downregulation might permit tumour cells to escape from host immune surveillance [26].

**Table 1:** 20 most frequently selected genes of Hepatocellular Carcinoma data selected by LOOCSFS

Gene no.	Freq. of selection	Description
X03100	198	HLA-SB alpha gene (class II antigen) extracted from Human HLA-SB(DP) alpha gene
M33600	196	Human MHC class II HLA-DR-beta-1 (HLA-DRB1) mRNA
X16663	194	Human HS1 gene for heamatopoietic lineage cell specific protein
U19713	193	Human allograft-inflammatory factor-1 mRNA
L36033	193	Human pre-B cell stimulating factor homologue (SDF1b) mRNA
X00274	192	Human gene for HLA-DR alpha heavy chain a class II antigen (immune response gene) of the major histocompatibility complex (MHC)
L08895	191	Homo sapiens MADS/MEF2-family transcription factor (MEF2C) mRNA
X15341	190	Human COX VIa-L mRNA for cytochrome c oxidase liver-specific subunit VIa (EC 1.9.3.1)
M59465	190	Human tumor necrosis factor alpha inducible protein A20 mRNA
HG1872-HT1907	190	Major Histocompatibility Complex, Dg
L11695	189	Human activin receptor-like kinase (ALK-5) mRNA
Y10032	185	H.sapiens mRNA for putative serine/threonine protein kinase
M13560	184	Human Ia-associated invariant gamma-chain gene
L76927	182	Human galactokinase (GALK1) gene
X16323	181	Human mRNA for hepatocyte growth factor (HGF)
U69546	181	Human RNA binding protein Etr-3 mRNA
AB000409	180	Human mRNA for MNK1
X75042	177	H.sapiens rel proto-oncogene mRNA
HG3576-HT3779	175	Major Histocompatibility Complex, Class Ii Beta W52
M87503	175	Human IFN-responsive transcription factor subunit mRNA

**Table 2:** 20 most frequently selected genes of Hepatocellular Carcinoma data selected by GLGS

Gene no.	Freq. of selection	Description
AB000409	128	Human mRNA for MNK1
L11695	109	Human activin receptor-like kinase (ALK-5) mRNA
X15341	105	Human COX VIa-L mRNA for cytochrome c oxidase liver-specific subunit VIa (EC 1.9.3.1)
U79294	105	Human clone 23748 mRNA
Y10032	103	H.sapiens mRNA for putative serine/threonine protein kinase
L76927	103	Human galactokinase (GALK1) gene
D28915	80	Human gene for hepatitis C-associated microtubular aggregate protein p44
L08895	79	Homo sapiens MADS/MEF2-family transcription factor (MEF2C) mRNA
M64925	75	Human palmitoylated erythrocyte membrane protein (MPP1) mRNA
X75042	73	H.sapiens rel proto-oncogene mRNA
X58377	72	Human mRNa for adipogenesis inhibitory factor
M59465	70	Human tumor necrosis factor alpha inducible protein A20 mRNA
X15422	68	Human mRNA for mannose-binding protein C
D78335	66	Human mRNA for 5 -terminal region of UMK
U26710	64	Human cbl-b mRNA
L36033	64	Human pre-B cell stimulating factor homologue (SDF1b) mRNA
HG4063-HT4333	61	Transcription Factor Hbf-2
D90086	60	Human pyruvate dehydrogenase (EC 1.2.4.1) beta subunit gene, exons 10-Jan
U03105	59	Human B4-2 protein mRNA
L22343	58	Human nuclear phosphoprotein mRNA

#### Glioma dataset

In their original work, Nutt *et al*. identified 77 important genes that were used to construct a 20-gene *k*-NN classifier in a leave-one-out cross-validation procedure [3]. Tables 3 and 4 present the 20 most frequently selected genes by LOOCSFS and GLGS. We found from Tables 3 and 4 that the genes L39874 (Please note that according to the original work, L39874-630_at and L39784-631_g_at are two different features obtained by applying the same probe set to different regions of a same gene), AB007960 and D29643 may be very important since they are both frequently selected in the original work as well as by our two algorithms. In addition, the gene Y00815 is also identified by both LOOCSFS and GLGS algorithm. Hence, it may also be important.

**Table 3:** 20 most frequently selected genes of Glioma data selected by LOOCSFS

Gene no.	Freq. of selection	Description
zL39874 (630_at)	199	Homo sapiens deoxycytidylate deaminase gene
zL39874 (631_g_at)	199	Homo sapiens deoxycytidylate deaminase gene
U84007	195	Human glycogen debranching enzyme isoform 1 (AGL) mRNA
U84573	194	Homo sapiens lysyl hydroxylase isoform 2 (PLOD2) mRNA
AL079277	192	Homo sapiens mRNA full length insert cDNA clone
AB007960	191	chromosome 1 specific transcript KIAA0491
AF070546	190	Homo sapiens clone 24607 mRNA sequence
AB028964	189	Homo sapiens mRNA for KIAA1041 protein
AB026436	189	Homo sapiens mRNA for dual specificity phosphatase MKP-5
AB020684	189	Homo sapiens mRNA for KIAA0877 protein
M97388	188	Human TATA binding protein-associated phosphoprotein (DR1) mRNA
Y00815	185	Human mRNA for LCA-homolog. LAR protein (leukocyte antigen related)
AW006742	185	wr28g10.x1 Homo sapiens cDNA
D29643	185	Human mRNA for KIAA0115 gene
U42390	182	Homo sapiens Trio mRNA
W25874	181	14e9 Homo sapiens cDNA
Z98946	181	Human DNA sequence from clone 376D21 on chromosome Xq11.1–12
X82676	180	Homo sapiens mRNA for tyrosine phosphatase
Z35307	178	H.sapiens mRNA for endothelin-converting-enzyme
L13278	177	Homo sapiens zeta-crystallin/quinone reductase mRNA

**Table 4:** 20 most frequently selected genes of Glioma data selected by GLGS

Gene no.	Freq. of selection	Description
AB007960	118	chromosome 1 specific transcript KIAA0491
U65002	107	Human zinc finger protein PLAG1 mRNA
D29643	101	Human mRNA for KIAA0115 gene
AB007975	95	Homo sapiens mRNA, chromosome 1 specific transcript KIAA0506
L34075	92	Human FKBP-rapamycin associated protein (FRAP) mRNA
AJ010228	91	Homo sapiens mRNA for RET finger protein-like 1
M12625	90	Human lecithin-cholesterol acyltransferase mRNA
zL39874 (630_at)	86	Homo sapiens deoxycytidylate deaminase gene
zL39874 (631_g_at)	86	Homo sapiens deoxycytidylate deaminase gene
J00077	84	Human alpha-fetoprotein (AFP) mRNA
Y00815	83	Human mRNA for LCA-homolog. LAR protein (leukocyte antigen related)
AF013588	82	Homo sapiens mitogen-activated protein kinase kinase 7 (MKK7) mRNA
M73481	80	Human gastrin releasing peptide receptor (GRPR) mRNA
X52486	78	Human mRNA for uracil-DNA glycosylase
AB020678	76	Homo sapiens mRNA for KIAA0871 protein
AL031432	76	Human DNA sequence from clone 465N24 on chromosome 1p35.1–36.13
AB000275	73	Homo sapiens mRNA for DAP-2
U95044	72	Human zinc finger protein (FDZF2) mRNA
U25801	72	Human Tax1 binding protein mRNA
J05581	71	Human polymorphic epithelial mucin (PEM) mRNA

Another interesting observation on the two datasets is that a gene is usually less frequently selected by GLGS than by LOOCSFS, which indicates that the 200 gene subsets selected by GLGS are more different from one another (more diverse) than the gene subsets selected by LOOCSFS. Since the training sets for the 200 bootstrap samples are different, the optimal gene subsets for classification are also expected to be different. Hence, this observation is consistent with the fact that GLGS can select gene subsets leading to lower generalization performance as seen in figures 1 and 2.

## Discussion

In practice, choosing a gene selection algorithm for discriminant analysis usually depends on the problem involved. According to the presented experimental results, if only one gene subset that leads to the lowest generalization error is needed, the GLGS is more appealing. However, in some cases we have to trade some accuracy for efficiency. Among the seven discussed gene selection methods, the marginal filter approach is the most efficient, but it generates the highest generalization error. If *d*<1000 and *t*<100, the GLGS algorithm is more time consuming than the approaches employing the sequential forward selection schemes. In this case, if one wants to obtain the solution faster and also achieve higher accuracy than a marginal filter method, the LOOCSFS and SVM-RFE may be more suitable than the GLGS. If the number of samples is relatively larger than the number of genes, which is quite unlikely for microarray data, the MAHSFS may be a better choice. Finally, the GLGS algorithm appears to be a good choice for small number of samples with large *d *and *t*, which is true for most microarray-based gene selection scenarios.

If the LOOCSFS or GLGS is chosen to carry out gene selection, one needs to define the number of the genes to be selected. In our experiments, we set this number as 100 for both datasets, of course 100 may not be the optimal number for achieving lowest generalization error on all microarray datasets and in practice one may need to estimate the optimal one for different datasets. Although our aim in this paper is not to investigate this point thoroughly, some suggestions can be found in previous studies [14]. One approach is to terminate the selection procedure when a given criterion does not improve significantly when more genes are incorporated. This strategy can be easily included in the program so that the algorithm can terminate automatically, and the criterion can be the LOOC measure, or simply the cross-validation error. Another approach is selecting a sufficiently large number of genes. Then by looking at the curve of generalization error, a human expert can determine the optimal number of genes to be selected so that the generalization error does not decrease significantly when more genes are selected. Based on the last approach and the experimental results presented in figures 1 and 2, we are further able to recommend some choices of the number of selected genes for the discussed two datasets. Our recommendation is based on the results of GLGS algorithm since it achieves the lowest external b.632+ error on the two datasets. For the Hepatocellular Carcinoma dataset, after selecting 40 features, including more genes can only result in no more than 1% reduction in the external b.632+ error. If 40 is a relatively large number for a gene subset, we can select 20 genes. It can be observed from figure 1 that the error increases significantly when the number of selected genes is less than 20. Therefore, we recommend 20 and 40 as two choices for the Hepatocellular Carcinoma dataset. Similarly, we can find from figure 2 that 15 and 30 can be good choices for the glioma dataset.

To identify important genes and study the possible interactions between them, one may need to select a number of different gene subsets that can all solve the classification problem with similar high accuracy. For this purpose, we feel that not only different training data (as in [23]), but also different selection algorithms should also be employed to render a comprehensive exploration of the useful genes. In this case, our methods can be used together with many other approaches. In the context of machine learning, this approach is referred as an ensembling, which has also been used to the gene selection problems. It should be noted that generally any algorithm that selects a single gene subset could be used as a component of such an ensemble system. Therefore, our work can be viewed as providing new choices to build an ensemble system.

## Conclusion

In this study, we have proposed two gene selection algorithms, the LOOCSFS and the GLGS algorithms based on an efficient and exact calculation of the leave-one-out cross-validation error of LS-SVM. The GLGS algorithm is different from traditional gene selection algorithms for that it solves the involved optimization problem in a much lower dimensional space, thus significantly reduces the computational cost of the selection procedure, while still GLGS selects genes from the original gene set. As both LOOCSFS and GLGS algorithms are derived from the exact calculation of leave-one-out cross-validation error, they are promising to select gene subsets leading to low generalization error. Experimental results show that the GLGS is also more efficient than traditional algorithms when the microarray data are represented by a large number of genes or a large number of genes to be selected from the whole gene set. Furthermore, our algorithms can be easily incorporated into more sophisticated ensemble systems to enhance overall gene selection performance.

## Methods

### Least square support vector machines

Belonging to the large family of so-called kernel methods [27], the least squares support vector machine (LS-SVM) [28,29] is a modification of the standard support vector machine. Suppose we are given *n *training sample pairs {**x**_*i*_, y_*i*_} where **x**_*i *_is a *d*-dimensional column vector representing the *i*th sample, and y_*i *_is the class label of **x**_*i*_, which is either +1 or -1. The LS-SVM employs a set of mapping functions Ö to map the data into a reproducing kernel Hilbert space (RKHS), and performs classification in it. Using the kernel function *k*(**x**_*i*_, **x**_*j*_) = **x**_*i*_^*T*^**x**_*j*_, the linear decision boundary of the LS-SVM can be formulated as

**w**^*T*^**x**+*b *= 0     (1)

where **w **= [*w1*, *w2*, ..., *wn*]^*T *^and *b *is a scalar. **w **and *b *can be obtained by solving the optimization problem:

min⁡w,eJ(w,e)=12wTw+γ2∑i=1nei2     (2)
 MathType@MTEF@5@5@+=feaafiart1ev1aaatCvAUfKttLearuWrP9MDH5MBPbIqV92AaeXatLxBI9gBaebbnrfifHhDYfgasaacH8akY=wiFfYdH8Gipec8Eeeu0xXdbba9frFj0=OqFfea0dXdd9vqai=hGuQ8kuc9pgc9s8qqaq=dirpe0xb9q8qiLsFr0=vr0=vr0dc8meaabaqaciaacaGaaeqabaqabeGadaaakeaadaWfqaqaaiGbc2gaTjabcMgaPjabc6gaUbWcbaWexLMBbXgBcf2CPn2qVrwzqf2zLnharyGvLjhzH5wyaGabbiaa=DhacaWFSaGaa8xzaaqabaGccqWGkbGscqGGOaakcaWF3bGaa8hlaiaa=vgacqGGPaqkcqGH9aqpdaWcaaqaaiabigdaXaqaaiabikdaYaaacaWF3bWaaWbaaSqabeaacqWGubavaaGccaWF3bGaey4kaSYaaSaaaeaacqaHZoWzaeaacqaIYaGmaaWaaabCaeaacqWGLbqzdaqhaaWcbaGaemyAaKgabaGaeGOmaidaaaqaaiabdMgaPjabg2da9iabigdaXaqaaiabd6gaUbqdcqGHris5aOGaaCzcaiaaxMaadaqadaqaaiabikdaYaGaayjkaiaawMcaaaaa@5BE0@

*s.t.y*_*i*_(**w**^*T*^**x**_*i*_+*b*) = 1-*e*_*i *_    (3)

where *e*_*i *_denotes regression error for sample **x**_*i*_, **e **= [*e*_1_, *e*_2_, ..., *e*_*n*_] and γ is a given positive constant introduced to adjust the compromise between generalization and training errors. After introducing Lagrangian multipliers, the optimization problem can be converted to the following linear system:

[0YTYΩ+γ−1I][bα]=[0→1]     (4)
 MathType@MTEF@5@5@+=feaafiart1ev1aaatCvAUfKttLearuWrP9MDH5MBPbIqV92AaeXatLxBI9gBaebbnrfifHhDYfgasaacH8akY=wiFfYdH8Gipec8Eeeu0xXdbba9frFj0=OqFfea0dXdd9vqai=hGuQ8kuc9pgc9s8qqaq=dirpe0xb9q8qiLsFr0=vr0=vr0dc8meaabaqaciaacaGaaeqabaqabeGadaaakeaadaWadaqaauaabeqaciaaaeaacqaIWaamaeaatCvAUfeBSjuyZL2yd9gzLbvyNv2CaeHbwvMCKfMBHbaceeGaa8xwamaaCaaaleqabaGaemivaqfaaaGcbaGaa8xwaaqaaGGabiab+L6axjabgUcaRiabeo7aNnaaCaaaleqabaGaeyOeI0IaeGymaedaaGqabOGae0xsaKeaaaGaay5waiaaw2faamaadmaabaqbaeqabiqaaaqaaiabdkgaIbqaaiab+f7aHbaaaiaawUfacaGLDbaacqGH9aqpdaWadaqaauaabeqaceaaaeaadaWfqaqaaiabicdaWaWcbaGamaiMgkziUcqabaaakeaacaWFXaaaaaGaay5waiaaw2faaiaaxMaacaWLjaWaaeWaaeaacqaI0aanaiaawIcacaGLPaaaaaa@552F@

where **Y **= [*y*_1_, *y*_2_, ..., *y*_*n*_] ^*T*^, **Ù **= {y_*i*_y_*j*_**x**_*i *_^*T*^**x***j*}, 1→
 MathType@MTEF@5@5@+=feaafiart1ev1aaatCvAUfKttLearuWrP9MDH5MBPbIqV92AaeXatLxBI9gBaebbnrfifHhDYfgasaacH8akY=wiFfYdH8Gipec8Eeeu0xXdbba9frFj0=OqFfea0dXdd9vqai=hGuQ8kuc9pgc9s8qqaq=dirpe0xb9q8qiLsFr0=vr0=vr0dc8meaabaqaciaacaGaaeqabaqabeGadaaakeaadaWfGaqaamXvP5wqSXMqHnxAJn0BKvguHDwzZbqegyvzYrwyUfgaiqqacaWFXaaaleqabaGaeyOKH4kaaaaa@3A20@ = [1, 1, ..., 1]^*T*^, **a **= [*α*_1_, *α*_2_, ..., *α*_*n*_]^*T*^and **I **is the identity matrix. Similar to the standard SVM, given a testing sample **x**, the discriminant function of the LS-SVM takes the form:

w=∑i=1nαiyixi     (5)
 MathType@MTEF@5@5@+=feaafiart1ev1aaatCvAUfKttLearuWrP9MDH5MBPbIqV92AaeXatLxBI9gBaebbnrfifHhDYfgasaacH8akY=wiFfYdH8Gipec8Eeeu0xXdbba9frFj0=OqFfea0dXdd9vqai=hGuQ8kuc9pgc9s8qqaq=dirpe0xb9q8qiLsFr0=vr0=vr0dc8meaabaqaciaacaGaaeqabaqabeGadaaakeaatCvAUfeBSjuyZL2yd9gzLbvyNv2CaeHbwvMCKfMBHbaceeGaa83Daiabg2da9maaqahabaGaeqySde2aaSbaaSqaaiabdMgaPbqabaGccqWG5bqEdaWgaaWcbaGaemyAaKgabeaakiaa=HhadaWgaaWcbaGaemyAaKgabeaaaeaacqWGPbqAcqGH9aqpcqaIXaqmaeaacqWGUbGBa0GaeyyeIuoakiaaxMaacaWLjaWaaeWaaeaacqaI1aqnaiaawIcacaGLPaaaaaa@4CAE@

f(x)=∑i=1nαiyixTxi+b     (6)
 MathType@MTEF@5@5@+=feaafiart1ev1aaatCvAUfKttLearuWrP9MDH5MBPbIqV92AaeXatLxBI9gBaebbnrfifHhDYfgasaacH8akY=wiFfYdH8Gipec8Eeeu0xXdbba9frFj0=OqFfea0dXdd9vqai=hGuQ8kuc9pgc9s8qqaq=dirpe0xb9q8qiLsFr0=vr0=vr0dc8meaabaqaciaacaGaaeqabaqabeGadaaakeaacqWGMbGzcqGGOaaktCvAUfeBSjuyZL2yd9gzLbvyNv2CaeHbwvMCKfMBHbaceeGaa8hEaiabcMcaPiabg2da9maaqahabaGaeqySde2aaSbaaSqaaiabdMgaPbqabaGccqWG5bqEdaWgaaWcbaGaemyAaKgabeaakiaa=HhadaahaaWcbeqaaiabdsfaubaakiaa=HhadaWgaaWcbaGaemyAaKgabeaakiabgUcaRiabdkgaIjaaxMaacaWLjaWaaeWaaeaacqaI2aGnaiaawIcacaGLPaaaaSqaaiabdMgaPjabg2da9iabigdaXaqaaiabd6gaUbqdcqGHris5aaaa@5453@

and the sign of *f *(**x**) is taken as the class label of **x**.

The main difference between the standard SVM and the LS-SVM is that for the standard SVM the equality constraints in Eq. (3) are replaced by inequality constraints, thus SVM involves solving a quadratic programming (QP) problem, which requires more expensive computation than solving a linear system. On the other hand, according to an empirical study [29], the LS-SVM is capable of achieving comparable performance as the standard SVM on many real-world problems. It has also achieved satisfactory classification accuracy on microarray data [17].

### The LOOC gene selection criterion

As mentioned before, a good evaluation criterion for feature selection should guarantee low generalization error and can be computed efficiently. Although the B.632+ technique has been proven to be the best estimator of generalization error [18], computing B.632+ error for every candidate gene subset is computationally too costly. Therefore, the B.632+ error is seldom employed as the evaluation criterion during the gene selection process. On the other hand, as it is proven that the leave-one-out cross-validation error (LOOE) is an almost unbiased estimator of the generalization error [30], and it can be easily computed as we will demonstrate in Eq. (9), it is acceptable to use LOOE as the evaluation criterion for feature selection.

Basically,, the direct calculation of the LOOE requires repeating the whole training procedure for *n *times, where *n *is the number of training samples. This is still time consuming. To simplify the calculation of LOOE for the standard SVM, several approaches have been discussed [31,32]. These approaches generally require training the SVM only once with the whole training set. Many of them are later extended as evaluation criteria for feature selection [14]. But, determined by the nature of SVM, all these approaches involve solving the QP problem, which still requires expensive computation. In the context of LS-SVM, Cawley and Talbot in [33] and Van Gestel *et al*. in [34] showed that the leave-one-out error of an LS-SVM can be efficiently and exactly evaluated. This approach is then successfully implemented in the LS-SVMlab toolbox [35]. Since the LS-SVM can be implemented more efficiently, we focus on the LS-SVM in this paper. First of all, an alternative efficient calculation of the LOOE of the LS-SVM is presented as our starting point based on the Lemma below:

#### Lemma 1

Given *n *training samples, let **w**_*i *_and *b*_*i *_denote the **w **and *b *achieved by training the LS-SVM after sample **x**_*i *_is removed, and denote the testing result of sample **x**_*i *_in the leave-one-out procedure as:

fi(x)=yi(wiTxi+bi)     (7)
 MathType@MTEF@5@5@+=feaafiart1ev1aaatCvAUfKttLearuWrP9MDH5MBPbIqV92AaeXatLxBI9gBaebbnrfifHhDYfgasaacH8akY=wiFfYdH8Gipec8Eeeu0xXdbba9frFj0=OqFfea0dXdd9vqai=hGuQ8kuc9pgc9s8qqaq=dirpe0xb9q8qiLsFr0=vr0=vr0dc8meaabaqaciaacaGaaeqabaqabeGadaaakeaacqWGMbGzdaahaaWcbeqaaiabdMgaPbaakiabcIcaOmXvP5wqSXMqHnxAJn0BKvguHDwzZbqegyvzYrwyUfgaiqqacaWF4bGaeiykaKIaeyypa0JaemyEaK3aaSbaaSqaaiabdMgaPbqabaGcdaqadaqaaiaa=DhadaqhaaWcbaGaemyAaKgabaGaemivaqfaaOGaa8hEamaaBaaaleaacqWGPbqAaeqaaOGaey4kaSIaemOyai2aaSbaaSqaaiabdMgaPbqabaaakiaawIcacaGLPaaacaWLjaGaaCzcamaabmaabaGaeG4naCdacaGLOaGaayzkaaaaaa@5033@

Then Eq. (8) holds:

*y*_*i*_*f*^*i*^(**x**) = 1-α_*i*_/(**H**^-1^)_*ii *_    (8)

where H=[K+γ−1I1→1→T0]
 MathType@MTEF@5@5@+=feaafiart1ev1aaatCvAUfKttLearuWrP9MDH5MBPbIqV92AaeXatLxBI9gBaebbnrfifHhDYfgasaacH8akY=wiFfYdH8Gipec8Eeeu0xXdbba9frFj0=OqFfea0dXdd9vqai=hGuQ8kuc9pgc9s8qqaq=dirpe0xb9q8qiLsFr0=vr0=vr0dc8meaabaqaciaacaGaaeqabaqabeGadaaakeaatCvAUfeBSjuyZL2yd9gzLbvyNv2CaeHbwvMCKfMBHbaceeGaa8hsaiabg2da9maadmaabaqbaeqabiGaaaqaaiaa=TeacqGHRaWkcqaHZoWzdaahaaWcbeqaaiabgkHiTiabigdaXaaakiaa=LeaaeaadaWfGaqaaiaa=fdaaSqabeaacqGHsgIRaaaakeaadaWfGaqaaiaa=fdaaSqabeaacqGHsgIRaaGcdaahaaWcbeqaaiabdsfaubaaaOqaaiabicdaWaaaaiaawUfacaGLDbaaaaa@4976@, **K **= {**x**_*i *_^*T*^**x**_*j*_} is the kernel matrix, and (**H**^-1^)_*ii *_denotes the *i*th diagonal element of the matrix **H**^-1 ^(The proof of Lemma 1 is available in Additional file 1). An exact calculation of the LOOE of LS-SVM can be derived from Eq. (8) as:

LOOE=12n[n−∑i=1nsign(1−αi/(H−1))ii]     (9)
 MathType@MTEF@5@5@+=feaafiart1ev1aaatCvAUfKttLearuWrP9MDH5MBPbIqV92AaeXatLxBI9gBaebbnrfifHhDYfgasaacH8akY=wiFfYdH8Gipec8Eeeu0xXdbba9frFj0=OqFfea0dXdd9vqai=hGuQ8kuc9pgc9s8qqaq=dirpe0xb9q8qiLsFr0=vr0=vr0dc8meaabaqaciaacaGaaeqabaqabeGadaaakeaacqWGmbatcqWGpbWtcqWGpbWtcqWGfbqrcqGH9aqpdaWcaaqaaiabigdaXaqaaiabikdaYiabd6gaUbaadaWadaqaaiabd6gaUjabgkHiTmaaqahabaGaem4CamNaemyAaKMaem4zaCMaemOBa42aaeWaaeaacqaIXaqmcqGHsislcqaHXoqydaWgaaWcbaGaemyAaKgabeaakiabc+caViabcIcaOmXvP5wqSXMqHnxAJn0BKvguHDwzZbqegyvzYrwyUfgaiqqacaWFibWaaWbaaSqabeaacqGHsislcqaIXaqmaaGccqGGPaqkaiaawIcacaGLPaaadaWgaaWcbaGaemyAaKMaemyAaKgabeaaaeaacqWGPbqAcqGH9aqpcqaIXaqmaeaacqWGUbGBa0GaeyyeIuoaaOGaay5waiaaw2faaiaaxMaacaWLjaWaaeWaaeaacqaI5aqoaiaawIcacaGLPaaaaaa@6392@

**Figure 5:**
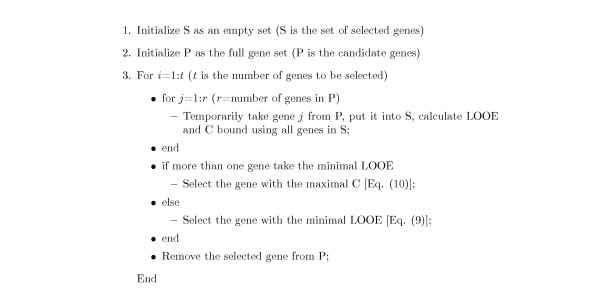
The LOOCSFS gene selection algorithm.

It should be noted that although they take different forms, Eq. (9) and the works presented in [33] and [34] generally share equivalent performance and property. Eq. (9) itself can be directly employed as the evaluation criterion for gene selection. But for a microarray dataset, which usually contains only a small number of samples, it is very likely that many candidate feature subsets may provide the same LOOE. Hence, based on Eq. (8), we propose the C bound as the supplementary criterion of Eq. (9):

C=∑i=1n(1−αi/(H−1)ii)−     (10)
 MathType@MTEF@5@5@+=feaafiart1ev1aaatCvAUfKttLearuWrP9MDH5MBPbIqV92AaeXatLxBI9gBaebbnrfifHhDYfgasaacH8akY=wiFfYdH8Gipec8Eeeu0xXdbba9frFj0=OqFfea0dXdd9vqai=hGuQ8kuc9pgc9s8qqaq=dirpe0xb9q8qiLsFr0=vr0=vr0dc8meaabaqaciaacaGaaeqabaqabeGadaaakeaacqWGdbWqcqGH9aqpdaaeWbqaamaabmaabaGaeGymaeJaeyOeI0IaeqySde2aaSbaaSqaaiabdMgaPbqabaGccqGGVaWlcqGGOaaktCvAUfeBSjuyZL2yd9gzLbvyNv2CaeHbwvMCKfMBHbaceeGaa8hsamaaCaaaleqabaGaeyOeI0IaeGymaedaaOGaeiykaKYaaSbaaSqaaiabdMgaPjabdMgaPbqabaaakiaawIcacaGLPaaadaWgaaWcbaGaeyOeI0cabeaaaeaacqWGPbqAcqGH9aqpcqaIXaqmaeaacqWGUbGBa0GaeyyeIuoakiaaxMaacaWLjaWaaeWaaeaacqaIXaqmcqaIWaamaiaawIcacaGLPaaaaaa@54FF@

where (x)_- _= min(0, x). Eq. (10) is motivated by the following consideration: A sample **x**_*i *_is misclassified in the leave-one-out procedure if *yif*^*i*^(**x**_*i*_) is negative, and absolute value of *yif*^*i*^(**x**_*i*_) indicates how close this sample is to the decision boundary. Therefore, for those samples misclassified in the LOO procedure (i.e. *yif*^*i*^(**x**_*i*_) is negative), a small absolute value of the term *yif*^*i*^(**x**_*i*_) is more preferable. Because a small value means that the sample is close to the decision boundary and might be classified correctly with a few more training data, while a large absolute value indicates that it is difficult to classify even if we are given more training data. By combining Eq. (9) with Eq. (10), we can now obtain the LOOC measure for the gene selection problem. The optimal gene subset for dicriminant analysis is the one leading to the smallest LOOE, if the same LOOE can be achieved on several gene subsets, the one with the largest value of the C bound is preferred (note that the C bound only requires negligible additional computation since the term 1-*α*_*i*_/(**H**^-1^)_*ii *_has been computed when calculating the LOOE). The advantage of the LOOC measure is that it is derived from the leave-one-out procedure and therefore is expected to be an accurate estimator of the generalization error. Further, LOOC measure can be calculated by training the LS-SVM with the whole training set only once, which requires solving a linear system and is much easier than solving a QP problem. Hence, the LOOC measure can be calculated even more efficiently than those SVM-based criteria [3,7]. By combining the LOOC measure with the sequential forward selection scheme, we propose the LOOCSFS gene selection algorithm, which is described in Figure 5.

### The gradient-based leave-one-out gene selection algorithm

In addition to the sequential forward selection, sequential floating forward selection, sequential backward elimination and sequential floating backward elimination search schemes, a possible alternative search scheme is the gradient descent method. Using gradient descent is not a totally new idea in the literature of the standard SVM. Chapelle *et al*. [32] employed the gradient descent approach to choose parameters for the standard SVM, they also suggested using the same framework to address feature selection problem. But the resultant algorithm requires using gradient descent to repeatedly solve an optimization problem, whose dimensionality is the same as the total number of genes. As the number of genes is usually huge in microarray data, this framework will be very time consuming for gene selection problems. Considering the specific properties of microarray data, we propose in this subsection a novel gene selection algorithm, named gradient-based leave-one-out gene selection (GLGS) algorithm. It is also based on the exact calculation of the LOOE of LS-SVM, and employs a gradient descent approach to optimize the evaluation criterion.

As we would like to use a gradient approach to optimize the evaluation criterion, the criterion must be differentiable, whereas both Eq. (9) and Eq. (10) are not. Hence, to obtain a differentiable criterion, the logistic LOOC (LLOOC) measure is proposed by modifying Eq. (9) as:

LLOOC=1n∑i=1n11+exp⁡(1−αi/(H−1)ii)     (11)
 MathType@MTEF@5@5@+=feaafiart1ev1aaatCvAUfKttLearuWrP9MDH5MBPbIqV92AaeXatLxBI9gBaebbnrfifHhDYfgasaacH8akY=wiFfYdH8Gipec8Eeeu0xXdbba9frFj0=OqFfea0dXdd9vqai=hGuQ8kuc9pgc9s8qqaq=dirpe0xb9q8qiLsFr0=vr0=vr0dc8meaabaqaciaacaGaaeqabaqabeGadaaakeaacqWGmbatcqWGmbatcqWGpbWtcqWGpbWtcqWGdbWqcqGH9aqpdaWcaaqaaiabigdaXaqaaiabd6gaUbaadaaeWbqaamaalaaabaGaeGymaedabaGaeGymaeJaey4kaSIagiyzauMaeiiEaGNaeiiCaa3aaeWaaeaacqaIXaqmcqGHsislcqaHXoqydaWgaaWcbaGaemyAaKgabeaakiabc+caViabcIcaOmXvP5wqSXMqHnxAJn0BKvguHDwzZbqegyvzYrwyUfgaiqqacaWFibWaaWbaaSqabeaacqGHsislcqaIXaqmaaGccqGGPaqkdaWgaaWcbaGaemyAaKMaemyAaKgabeaaaOGaayjkaiaawMcaaaaaaSqaaiabdMgaPjabg2da9iabigdaXaqaaiabd6gaUbqdcqGHris5aOGaaCzcaiaaxMaadaqadaqaaiabigdaXiabigdaXaGaayjkaiaawMcaaaaa@61EE@

The logistic function 1/(1+exp(x)) is commonly used to transfer output of an SVM-type classifier into a specific region [36]. Different from the C bound, the LLOOC measure ranges between (0,1). Hence it can be viewed as a probability that represents the generalization error of a classifier and can be useful for possible post-processing procedure. More precisely, large positive value of the term 1-*α*_*i*_/(**H**^-1^)_*ii *_generally corresponds to a small LOOE and LLOOC. Therefore, the genes can be evaluated by minimizing the LLOOC measure.

In the present study, to design an LS-SVM based gene selection algorithm, we introduce a diagonal matrix **V**, whose diagonal elements are scaling factors {*v*_1_, *v*_2_, ..., *v*_*d*_}, into the kernel matrix **K **to modify *k*(**x**_*i*_, **x**_*j*_) = **x**_*i*_^*T*^**x**_*j *_as *k*(**x**_*i*_, **x**_*j*_) = **x**_*i*_^*T*^**Vx**_*j*_. Consequently, the LLOOC can be viewed as a function of these scaling factors, and we can optimize the scaling factors by solving the *d*-dimensional minimization problem below:

#### Problem 1

Given a *d*-by-*d *diagonal matrix **V**, whose diagonal elements are the scaling factors {*v*_1_, *v*_2_, ..., *v*_*d*_}, minimize the LLOOC measure with respect to **V**.

For the optimized scaling factors, a smaller absolute value indicates that the corresponding gene is less important for achieving the minimal LLOOC measure and thereby low generalization error. Hence, genes can be selected according to the absolute value of the scaling factors.

Given the above-described problem, we can observe that the LLOOC measure is differentiable, and the partial derivative of it with respect to a scaling factor *v*_*k *_can be calculated by (Detailed derivations are available in Additional file 1):

∂LLOOC∂vk=1n(H−1)ii2∑i=1nexp⁡(1−αi/(H−1)ii)[1+exp⁡(1−αi/(H−1)ii)]2.[αi(H−1[∂K∂vk000]H−1)ii−Hii−1(M−1[∂Ω∂vk000]M−1[1→0])i]     (12)
 MathType@MTEF@5@5@+=feaafiart1ev1aaatCvAUfKttLearuWrP9MDH5MBPbIqV92AaeXatLxBI9gBaebbnrfifHhDYfgasaacH8akY=wiFfYdH8Gipec8Eeeu0xXdbba9frFj0=OqFfea0dXdd9vqai=hGuQ8kuc9pgc9s8qqaq=dirpe0xb9q8qiLsFr0=vr0=vr0dc8meaabaqaciaacaGaaeqabaqabeGadaaakeaafaqadeGabaaabaWaaSaaaeaacqGHciITcqWGmbatcqWGmbatcqWGpbWtcqWGpbWtcqWGdbWqaeaacqGHciITcqWG2bGDdaWgaaWcbaGaem4AaSgabeaaaaGccqGH9aqpdaWcaaqaaiabigdaXaqaaiabd6gaUnaabmaabaWexLMBbXgBcf2CPn2qVrwzqf2zLnharyGvLjhzH5wyaGabbiaa=HeadaahaaWcbeqaaiabgkHiTiabigdaXaaaaOGaayjkaiaawMcaamaaDaaaleaacqWGPbqAcqWGPbqAaeaacqaIYaGmaaaaaOWaaabCaeaadaWcaaqaaiGbcwgaLjabcIha4jabcchaWnaabmaabaGaeGymaeJaeyOeI0IaeqySde2aaSbaaSqaaiabdMgaPbqabaGccqGGVaWlcqGGOaakcaWFibWaaWbaaSqabeaacqGHsislcqaIXaqmaaGccqGGPaqkdaWgaaWcbaGaemyAaKMaemyAaKgabeaaaOGaayjkaiaawMcaaaqaamaadmaabaGaeGymaeJaey4kaSIagiyzauMaeiiEaGNaeiiCaa3aaeWaaeaacqaIXaqmcqGHsislcqaHXoqydaWgaaWcbaGaemyAaKgabeaakiabc+caViabcIcaOiaa=HeadaahaaWcbeqaaiabgkHiTiabigdaXaaakiabcMcaPmaaBaaaleaacqWGPbqAcqWGPbqAaeqaaaGccaGLOaGaayzkaaaacaGLBbGaayzxaaWaaWbaaSqabeaacqaIYaGmaaaaaaqaaiabdMgaPjabg2da9iabigdaXaqaaiabd6gaUbqdcqGHris5aOGaeiOla4YaamqaaeaacqaHXoqydaWgaaWcbaGaemyAaKgabeaakmaabmaabaGaa8hsamaaCaaaleqabaGaeyOeI0IaeGymaedaaOWaamWaaeaafaqabeGacaaabaWaaSaaaeaacqGHciITcaWFlbaabaGaeyOaIyRaemODay3aaSbaaSqaaiabdUgaRbqabaaaaaGcbaGaa8hmaaqaaiaa=bdaaeaacqaIWaamaaaacaGLBbGaayzxaaGaa8hsamaaCaaaleqabaGaeyOeI0IaeGymaedaaaGccaGLOaGaayzkaaWaaSbaaSqaaiabdMgaPjabdMgaPbqabaaakiaawUfaaaqaamaadiaabaGaeyOeI0Iaa8hsamaaDaaaleaacqWGPbqAcqWGPbqAaeaacqGHsislcqaIXaqmaaGcdaqadaqaaiaa=1eadaahaaWcbeqaaiabgkHiTiabigdaXaaakmaadmaabaqbaeqabiGaaaqaamaalaaabaGaeyOaIylcceGae4xQdCfabaGaeyOaIyRaemODay3aaSbaaSqaaiabdUgaRbqabaaaaaGcbaGaa8hmaaqaaiaa=bdaaeaacqaIWaamaaaacaGLBbGaayzxaaGaa8xtamaaCaaaleqabaGaeyOeI0IaeGymaedaaOWaamWaaeaafaqabeGabaaabaWaaCbiaeaacaWFXaaaleqabaGaeyOKH4kaaaGcbaaceaGaa0hmaaaaaiaawUfacaGLDbaaaiaawIcacaGLPaaadaWgaaWcbaGaemyAaKgabeaaaOGaayzxaaaaaiaaxMaacaWLjaWaaeWaaeaacqaIXaqmcqaIYaGmaiaawIcacaGLPaaaaaa@C17F@

where M=[Ω+γ−1IYYT0]
 MathType@MTEF@5@5@+=feaafiart1ev1aaatCvAUfKttLearuWrP9MDH5MBPbIqV92AaeXatLxBI9gBaebbnrfifHhDYfgasaacH8akY=wiFfYdH8Gipec8Eeeu0xXdbba9frFj0=OqFfea0dXdd9vqai=hGuQ8kuc9pgc9s8qqaq=dirpe0xb9q8qiLsFr0=vr0=vr0dc8meaabaqaciaacaGaaeqabaqabeGadaaakeaatCvAUfeBSjuyZL2yd9gzLbvyNv2CaeHbwvMCKfMBHbaceeGaa8xtaiabg2da9maadmaabaqbaeqabiGaaaqaaGGabiab+L6axjabgUcaRiabeo7aNnaaCaaaleqabaGaeyOeI0IaeGymaedaaOGaa8xsaaqaaiaa=LfaaeaacaWFzbWaaWbaaSqabeaacqWGubavaaaakeaacqaIWaamaaaacaGLBbGaayzxaaaaaa@4611@. Let **x**_*i *_= [*x*_*i1*_, *x*_*i2*_, ..., *x*_*id*_]^*T*^, then:

∂Ω∂vk={yiyjxikxjk} and ∂K∂vk={xikxjk}     (13)
 MathType@MTEF@5@5@+=feaafiart1ev1aaatCvAUfKttLearuWrP9MDH5MBPbIqV92AaeXatLxBI9gBaebbnrfifHhDYfgasaacH8akY=wiFfYdH8Gipec8Eeeu0xXdbba9frFj0=OqFfea0dXdd9vqai=hGuQ8kuc9pgc9s8qqaq=dirpe0xb9q8qiLsFr0=vr0=vr0dc8meaabaqaciaacaGaaeqabaqabeGadaaakeaadaWcaaqaaiabgkGi2IGabiab=L6axbqaaiabgkGi2kabdAha2naaBaaaleaacqWGRbWAaeqaaaaakiabg2da9maacmaabaGaemyEaK3aaSbaaSqaaiabdMgaPbqabaGccqWG5bqEdaWgaaWcbaGaemOAaOgabeaakiabdIha4naaBaaaleaacqWGPbqAcqWGRbWAaeqaaOGaemiEaG3aaSbaaSqaaiabdQgaQjabdUgaRbqabaaakiaawUhacaGL9baacqqGGaaicqqGHbqycqqGUbGBcqqGKbazcqqGGaaidaWcaaqaaiabgkGi2oXvP5wqSXMqHnxAJn0BKvguHDwzZbqegyvzYrwyUfgaiqqacaGFlbaabaGaeyOaIyRaemODay3aaSbaaSqaaiabdUgaRbqabaaaaOGaeyypa0ZaaiWaaeaacqWG4baEdaWgaaWcbaGaemyAaKMaem4AaSgabeaakiabdIha4naaBaaaleaacqWGQbGAcqWGRbWAaeqaaaGccaGL7bGaayzFaaGaaCzcaiaaxMaadaqadaqaaiabigdaXiabiodaZaGaayjkaiaawMcaaaaa@6DCC@

Therefore, we can solve the minimization problem by using a gradient descent approach. However, *d *is usually very large for microarray data, which means the Problem 1 is a high dimensional optimization problem in our case. As we have mentioned, the gradient descent approach takes a long time to converge for high dimensional optimization problem. To overcome this situation, in the GLGS algorithm, the scaling factors are not introduced into the original data directly. Instead, we first apply a principal component analysis (PCA) procedure to the microarray data to resolve the high dimensionality problem, and scaling factors are then introduced into the transformed data and optimized. In pattern recognition field, PCA is a commonly used approach for dimensionality reduction. Denoting the original high dimensional data by a *d*-by-*n *matrix **X**, PCA first computes a transformation matrix **T**, and then transforms **X **to a low dimensional space by **X**_*low *_= **TX**, where **X**_*low *_denotes the transformed data and is a *d*_*low*_-by-*n *matrix. Each feature of the transformed data is actually a linear combination of the features of original data (we refer the features of transformed data and original data as features and genes respectively), and most information of the original data can be preserved by setting the value of *d*_*low *_no larger than min(*d*, *n*) (specifically, we recommend to use *d = n *for the presented GLGS algorithm). In case of the microarray data analysis, because the number of samples is usually very small while the number of genes is huge, PCA can reduce dimensionality of the data significantly, which typically equals the number of samples. By this means, we only need to solve an optimization problem whose dimensionality is the number of samples, thereby reducing the computational cost.

**Figure 6:**
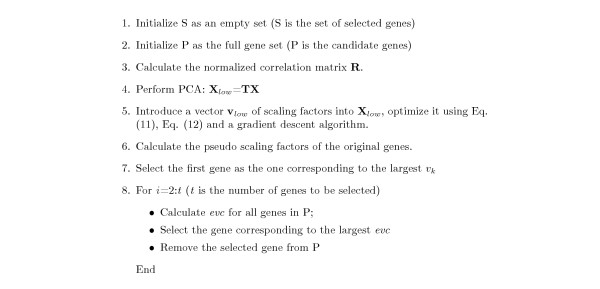
The GLGS gene selection algorithm.

After optimizing a *d*_*low *_dimensional vector **v**_*low *_of scaling factors in the transformed space, the scaling factors of the original genes, which are called pseudo scaling factors as they are not truly optimized, can be estimated based on three considerations: First of all, absolute values of the scaling factors of the transformed features indicate the importance of the transformed features for achieving the minimal LLOOC measure. Second, absolute values of the elements of **T **reveal how important the corresponding genes are for constructing the transformed data. Finally, correlation between genes plays an important part in gene selection problems. Hence it is usually expected that a set of uncorrelated genes are likely to be more informative. As a result, the pseudo scaling factors for the original genes can be estimated as:

**v **= **R**⌊*abs*(**T**^*T*^)⌋ [*abs*(**v**_*low*_)]     (14)

where **R **denotes the *d*-by-*d *correlation coefficient matrix of the original gene set, **v **= [v*1*, v*2*, ..., v*d*] ^*T *^and abs(**T**^*T*^) is the matrix whose elements take the absolute value of the elements of **T**^*T*^. The term ⌊*abs*(**T**^*T*^)⌋[*abs*(**v**_*low*_)] evaluates the genes' contribution for constructing the lower dimensional space. However, if two or more important genes are very similar to each other, they will have correspondingly large pseudo scaling factors while including all of them may not reduce the generalization error. In this case, we only need to select one of them as a representation and avoid selecting similar genes subsequently. Since the more a specific gene correlated to other genes, the better it can be viewed as a representation of many other genes, we combine the correlation matrix **R **with the term ⌊*abs*(**T**^*T*^)⌋[*abs*(**v**_*low*_)] in the final selection procedure. Given the scaling factors estimated for the original genes, we select genes sequentially from the original gene set based on the pseudo scaling factors and the *evc *criterion:

*evc *= (1-β)v_*k *_    (15)

where *β *is the largest correlation coefficient between *k*^*th *^gene and one of the already selected genes. Although large value of *v*_*k *_means the gene is possibly informative, large value of *β *indicates that the *k*th gene is highly correlated with at least one already selected gene. Hence, the term 1-*β *is introduced to control similarity between the selected genes. In each stage of the selection procedure, the gene with respect to the largest *evc *is the most desirable one and will be selected. The whole GLGS algorithm is described in Figure 6.

According to the definitions in [4], the GLGS algorithm can be categorized as an embedded method. Hence, it is more time consuming than a marginal filter method. The GLGS differs from previous wrapper and embedded approaches because it optimizes the evaluation criterion derived in a supervised manner in a transformed space with significantly reduced dimensions instead of the original space, while it selects genes from the original gene set based on results of the optimization. One main advantage of the GLGS over the other gene selection algorithms is scaling well to high dimensional data. In a gene selection algorithm, the evaluation criterion is computed repeatedly to assess candidate gene subsets. Hence, the computational cost of a gene selection algorithm is determined not only by computational complexity of the evaluation criterion, but also by the number of required evaluations. Although we have experimentally shown that GLGS can better scale to high dimensions and large number of selected genes, it is worth analyzing the computational complexity issue quantitatively. If the microarray data contain *d *genes and *t *of them are to be selected, the sequential forward selection and the sequential backward elimination schemes require (2*d*-*t*+1)*t*/2 and (2*d*-*t*-1)(*d*-*t*)/2 evaluations respectively. The sequential floating selection/elimination scheme requires more evaluations than the former two schemes and GAs generally requires even more evaluations than the floating schemes. As *d *and *t *increase, the number of evaluations required by these schemes will increase significantly. Since microarray data usually contain thousands of genes, all the traditional schemes are highly time consuming for the gene selection problem even if we employ an evaluation criterion that is easy to compute. In contrast to these traditional methods, because the computational complexity of Eq. (11) and Eq. (12) is mainly determined by the number of samples rather than the size of the whole gene set or the number of genes to be selected, time requirement of the GLGS algorithm will not increase much when *d *or *t *increases, as can be observed in Figure 3 and Figure 4. By performing minimization of Eq. (11) in the lower dimensional PC space, it requires much less evaluations for high dimensional data than the sequential forward selection, sequential floating forward selection, sequential backward elimination and sequential floating backward elimination schemes. For example, if 50 genes are to be selected from 5000, then the LOOCSFS algorithm requires 123775 evaluations, and SVM-RFE requires solving the QP problem for 4950 times because of its specific mechanism. For GLGS algorithm, the computational complexity is dominated by the PCA and gradient descent procedure. Generally, the gradient descent procedure can converge within 300 iterations. As the computational complexity of Eq. (11) and Eq. (12) is approximately two times of Eq. (9) and Eq. (10), the time requirement of the gradient descent procedure in this case is comparable to 600 evaluations of the LOOCSFS method, and less than 600 evaluations of SVM-RFE. The computational cost of the PCA procedure is a bit difficult to estimate, but our experimental results show that it can almost be neglected when *d *and *t *are large. Finally, as Eq. (11) is derived from the exact calculation of the LOOE of LS-SVM, GLGS also can select gene subsets leading to a low generalization error.

All the related proofs/derivations, the data used in the experiments and the programs of the LOOCSFS and GLGS algorithms are provided in the additional files.

## List of abbreviations

GA Genetic Algorithm

GLGS Gradient-based Leave-one-out Gene Selection

LLOOC Logistic LOOC

LOOC Leave-One-Out Calculation (measure)

LOOCSFS Gene selection method using LOOC measure and SFS scheme

LOOE Leave-One-Out cross-validation ErrorLSSFS Gene selection method using LS bound measure and SFS scheme

LSSFFS Gene selection method using LS bound measure and SFFS scheme

LS-SVM Least Squares Support Vector MachineMAHSFS Gene selection method using Mahalanobis measure and SFS scheme

PCA Principal Component Analysis

RFE Recursive Feature Elimination (sequential backward elimination)

SFS Sequential Forward Selection scheme

SFFS Sequential Floating Forward Selection scheme

SVM-RFE SVM-based Recursive Feature Elimination algorithm

## Authors' contributions

KT developed the LOOC criteria, coded all procedures and conducted experiments. PNS proposed the usage of correlation in the GLGS procedure. XY suggested the usage of gradient descent. All three authors participated in the preparation of the manuscript.

## Supplementary Material

Additional File 1 **The proof of Lemma 1 and derivations of Eq. (12)**. Additional file descriptions text (including details of how to view the file, if it is in a non-standard format). Click here for file
