# Supplementary material for: Gene selection algorithms for microarray data based on least squares support vector machine
Source: BMC Bioinformatics. 2006 Feb 27;7:95. doi: 10.1186/1471-2105-7-95 (PMC1409801; doi:10.1186/1471-2105-7-95)
Supplement: Additional File 1 — The proof of Lemma 1 and derivations of Eq. (12). Additional file descriptions text (including details of how to view the file, if it is in a non-standard format). [file 1471-2105-7-95-S1.pdf]

1 Notations

Note: all results of this proof is applicable to any kernel functions, but for simplicity we use k($\mathbf{x}_i$, $\mathbf{x}_j$)=$\mathbf{x}_i{}^T\mathbf{x}_i$.

Scalars: b, μ and γ

Vectors:

$\mathbf{x}_i$ is the ith sample, represented by a d-dimensional vector.
$\mathbf{Y}$=[y_1, y_2, ..., y_n]^T is the class label of training samples.
$\vec{\mathbf{1}}$=[1, 1, ..., 1]^T
$\mathbf{w}$, $\mathbf{e}$, $\boldsymbol{\alpha}^0$, $\boldsymbol{\alpha}^p$, $\boldsymbol{\beta}$, $\boldsymbol{\delta}$, $\boldsymbol{\lambda}$, $\boldsymbol{\lambda}^*$ and $\boldsymbol{\lambda}'$ are all n-dimensional column vectors. For example: $\mathbf{w}$=[w_1, w_2, ..., w_n]^T.
$\bar{\boldsymbol{\lambda}} = [\lambda_1, \lambda_2, \ldots, \lambda_{p-1}, \lambda_{p+1}, \ldots, \lambda n, \mu]^T$

Matrices:

$\mathbf{I}$ is the identity matrix.
$\mathbf{K}$=$\{\mathbf{x}_i{}^T\mathbf{x}_j\}$, $\bar{\mathbf{K}}$=$\mathbf{K}$+$\gamma^{-1}\mathbf{I}$, $\boldsymbol{\Omega}$=$\{y_i y_j \mathbf{x}_i{}^T\mathbf{x}_j\}$, $\bar{\boldsymbol{\Omega}}$=$\boldsymbol{\Omega}+\gamma^{-1}\mathbf{I}$

$$\mathbf{H}=\begin{bmatrix} \mathbf{K}+\gamma^{-1}\mathbf{I} & \vec{\mathbf{1}} \\ \vec{\mathbf{1}}^T & 0 \end{bmatrix}, \quad \mathbf{M}=\begin{bmatrix} \boldsymbol{\Omega}+\gamma^{-1}\mathbf{I} & \mathbf{Y} \\ \mathbf{Y}^T & 0 \end{bmatrix}$$

For a matrix $\mathbf{A}$, $\mathbf{A}_{ij}$ denotes the ith elements of the jth column of $\mathbf{A}$, $\mathbf{H}_{/ij}$ is the jth column of $\mathbf{A}$ with the ith entry removed and $\mathbf{A}_{/i}$ is the submatrix of $\mathbf{A}$ with row and column i removed.

2 A brief introduction to the LS-SVM

As have been introduced, the linear decision boundary of LS-SVM can be described as:

$$\mathbf{w}^T\mathbf{x} + b = 0 \tag{1}$$

and LS-SVM can be formulated as the optimization problem:

$$\min_{\mathbf{w},\mathbf{e}} J(\mathbf{w}, \mathbf{e}) = \frac{1}{2}\mathbf{w}^T\mathbf{w} + \frac{\gamma}{2}\mathbf{e}^T\mathbf{e} \tag{2}$$

$$s.t. \quad y_i[\mathbf{w}^T\mathbf{x}_i + b] = 1 - e_i \tag{3}$$

where $\mathbf{w}$=[w_1, w_2, ..., w_n]^T, e_i denotes regression error for sample $\mathbf{x}_i$, $\mathbf{e}$=[e_1, e_2, ..., e_n]^T, b is a scalar and γ is a given positive constant that is introduced to adjust the compromise between generalization and training error. After introducing Lagrangian multipliers, solution to this optimization problem is given by the saddle point of the Lagrangian:

$$L(\mathbf{w}, b, \mathbf{e}, \boldsymbol{\alpha}) = \frac{1}{2}\mathbf{w}^T\mathbf{w} + \frac{\gamma}{2}\mathbf{e}^T\mathbf{e} - \sum_{i=1}^{n}\alpha_i[y_i(\mathbf{w}^T\mathbf{x}_i + b) - 1 + e_i] \tag{4}$$

Since the conditions for optimality is the partial derivatives of L with respect to $\mathbf{w}$, b, e_i and α_i being zero, we can get

$$\mathbf{w} = \sum_{i=1}^{n} \alpha_i y_i \mathbf{x}_i, \quad \sum_{i=1}^{n} \alpha_i y_i = 0, \quad and \quad \alpha_i = \gamma e_i \tag{5}$$

Combine Eq. (3) with Eq. (5), solution of the LS-SVM can be computed by solving a linear system:

$$\begin{bmatrix} 0 & \mathbf{Y}^T \\ \mathbf{Y} & \mathbf{\Omega} + \gamma^{-1}\mathbf{I} \end{bmatrix} \begin{bmatrix} b \\ \boldsymbol{\alpha} \end{bmatrix} = \begin{bmatrix} 0 \\ \vec{\mathbf{1}} \end{bmatrix} \tag{6}$$

where $\mathbf{Y}$=$[y_1, y_2, \ldots, y_n]^T$, $\mathbf{\Omega}$=$\{y_i y_j {\mathbf{x}_i}^T \mathbf{x}_i\}$, $\vec{\mathbf{1}}$=$[1, 1, \ldots, 1]^T$, $\boldsymbol{\alpha}$=$[\alpha_1, \alpha_2, \ldots, \alpha_n]^T$ and $\mathbf{I}$ is the identity matrix. Further, re-substitute Eq. (3) and Eq. (5) to Eq. (4), Eq. (2) can be converted to another optimization problem:

$$maxL(\boldsymbol{\alpha}) = \sum_{i=1}^{n} \alpha_i - \frac{1}{2} \sum_{i,j=1}^{n} \alpha_i \alpha_j y_i y_j \mathbf{x}_i^T \mathbf{x}_j - \frac{1}{2} \sum_{i=1}^{n} \alpha_i^2 / \gamma \tag{7}$$

$$s.t. \quad \sum_{i=1}^{n} \alpha_i y_i = 0 \tag{8}$$

3 The proof of Lemma 1

When employing the entire training set to train the LS-SVM, we use $\mathbf{w}$, b, $\boldsymbol{\alpha}^0$ and α_i^0 to denote the corresponding $\mathbf{w}$, b, $\boldsymbol{\alpha}$ and α_i in the previous section. When the sample $\mathbf{x}_p$ is removed from the training set in the LOO procedure, these terms are denoted by $\mathbf{w}_p$, b_p, $\boldsymbol{\alpha}^p$ and α_i^p respectively. Then we have

$$\mathbf{w}_p = \sum_{i=1, i \neq p}^{n} \alpha_i^p y_i \mathbf{x}_i \tag{9}$$

$$f^p(\mathbf{x}_p) = \mathbf{w}_p^T \mathbf{x}_p + b_p \tag{10}$$

To prove the Lemma 1, we first propose another Lemma as below:

Lemma 2: For any training sample $\mathbf{x}_p$, the following equality holds:

$$1 - y_p f^p(\mathbf{x}_p) = \min_{\boldsymbol{\lambda}} \alpha_p^0 \boldsymbol{\lambda}^T (\mathbf{K} + \gamma^{-1}\mathbf{I}) \boldsymbol{\lambda} \tag{11}$$

where $\boldsymbol{\lambda}$=$[\lambda_1, \lambda_2 \ldots, \lambda_n]^T$, and $\mathbf{K}$=$\{{\mathbf{x}_i}^T \mathbf{x}_i\}$

$$s.t. \quad \lambda_p = -1, \quad \sum_{i=1}^{n} \lambda_i = 0 \tag{12}$$

Proof of Lemma 2: For the entire training set, $\boldsymbol{\alpha}^0$=$[\alpha_1^0, \alpha_2^0 \ldots, \alpha_n^0]^T$ maximizes Eq. (7) subject to Eq. (8). So we have

$$L(\boldsymbol{\alpha}^0) \geq L(\boldsymbol{\alpha}^p + \boldsymbol{\beta}) \tag{13}$$

where $\boldsymbol{\beta}$=$[\beta_1,\ \beta_2 \ldots,\ \beta_n]^T$ satisfies the following condition:

$$\sum_{i=1}^{n} \beta_i y_i = 0 \tag{14}$$

Similarly, When the LS-SVM is trained in the leave-one-out procedure and sample $\mathbf{x}_p$ is removed, the solution $\boldsymbol{\alpha}^p$=$[\alpha_1^p,\ \alpha_2^p \ldots,\ \alpha_n^p]^T$ maximizes Eq. (7) subject to Eq. (8) and an additional constraint:

$$\alpha_p^p = 0 \tag{15}$$

Therefore, the following inequality holds:

$$L(\boldsymbol{\alpha}^p) \geq L(\boldsymbol{\alpha}^0 - \boldsymbol{\delta}) \tag{16}$$

where $\boldsymbol{\delta}$=$[\delta_1,\ \delta_2 \ldots,\ \delta_n]^T$ satisfies the constraints:

$$\delta_p = \alpha_p^0, \quad \sum_{i=1}^{n} \delta_i y_i = 0 \tag{17}$$

We can obtain

$$L(\boldsymbol{\alpha}^p + \boldsymbol{\beta}) - L(\boldsymbol{\alpha}^p) \leq L(\boldsymbol{\alpha}^0) - L(\boldsymbol{\alpha}^p) \leq L(\boldsymbol{\alpha}^0) - L(\boldsymbol{\alpha}^0 - \boldsymbol{\delta}) \tag{18}$$

Let us denote $\mathbf{I}_1$=$L(\boldsymbol{\alpha}^p + \boldsymbol{\beta}) - L(\boldsymbol{\alpha}^p)$ and $\mathbf{I}_2$=$L(\boldsymbol{\alpha}^0) - L(\boldsymbol{\alpha}^0 - \boldsymbol{\delta})$ and calculate them seperately:

$$\begin{aligned}
\mathbf{I}_1 &= L(\boldsymbol{\alpha}^p + \boldsymbol{\beta}) - L(\boldsymbol{\alpha}^p) \\
&= \sum_{i=1}^{n}(\alpha_i^p + \beta_i) - \frac{1}{2}\sum_{i,j=1}^{n}(\alpha_i^p + \beta_i)(\alpha_j^p + \beta_j) y_i y_j \mathbf{x}_i^T \mathbf{x}_j - \frac{1}{2\gamma}\sum_{i=1}^{n}(\alpha_i^p + \beta_i)^2 \\
&\quad - \sum_{i=1}^{n} \alpha_i^p + \frac{1}{2}\sum_{i,j=1}^{n} \alpha_i^p \alpha_j^p y_i y_j \mathbf{x}_i^T \mathbf{x}_j + \frac{1}{2\gamma}\sum_{i=1}^{n}(\alpha_i^p)^2 \\
&= \sum_{i=1}^{n} \beta_i - \sum_{i,j=1}^{n} \alpha_i^p \beta_j y_i y_j \mathbf{x}_i^T \mathbf{x}_j - \frac{1}{2}\sum_{i,j=1}^{n} \beta_i \beta_j y_i y_j \mathbf{x}_i^T \mathbf{x}_j - \sum_{i=1}^{n} \alpha_i^p \beta_i/\gamma - \frac{1}{2}\sum_{i=1}^{n} \beta_i^2/\gamma \\
&= \sum_{j=1}^{n} \beta_j (1 - y_j \sum_{i=1}^{n} \alpha_i^p y_i \mathbf{x}_i^T \mathbf{x}_j - \alpha_j^p/\gamma) - \frac{1}{2}(\sum_{i,j=1}^{n} \beta_i \beta_j y_i y_j \mathbf{x}_i^T \mathbf{x}_j + \sum_{i=1}^{n} \beta_i^2/\gamma) \\
&= \sum_{j=1}^{n} \beta_j (1 - y_j \mathbf{w}_p^T \mathbf{x}_j - \alpha_j^p/\gamma) - \frac{1}{2}(\sum_{i,j=1}^{n} \beta_i \beta_j y_i y_j \mathbf{x}_i^T \mathbf{x}_j + \sum_{i=1}^{n} \beta_i^2/\gamma)
\end{aligned} \tag{19}$$

Since $\sum_{i=1}^{n} \beta_i y_i = 0$

$$\begin{aligned}
&\sum_{j=1}^{n} \beta_j (1 - y_j \mathbf{w}_p^T \mathbf{x}_j - \alpha_j^p/\gamma) - \frac{1}{2}(\sum_{i,j=1}^{n} \beta_i \beta_j y_i y_j \mathbf{x}_i^T \mathbf{x}_j + \sum_{i=1}^{n} \beta_i^2/\gamma) \\
&= \sum_{j=1}^{n} \beta_j (1 - y_j \mathbf{w}_p^T \mathbf{x}_j - \alpha_j^p/\gamma) - \frac{1}{2}(\sum_{i,j=1}^{n} \beta_i \beta_j y_i y_j \mathbf{x}_i^T \mathbf{x}_j + \sum_{i=1}^{n} \beta_i^2/\gamma) - \sum_{i=1}^{n} \beta_i y_i b_p
\end{aligned}$$

$$
\begin{aligned}
&= \sum_{j=1}^{n} \beta_j [1 - y_j(\mathbf{w}_p^T \mathbf{x}_j + b_p) - \alpha_j^p/\gamma] - \frac{1}{2}(\sum_{i,j=1}^{n} \beta_i \beta_j y_i y_j \mathbf{x}_i^T \mathbf{x}_j + \sum_{i=1}^{n} \beta_i^2/\gamma) \\
&= \beta_p [1 - y_p(\mathbf{w}_p^T \mathbf{x}_p + b_p)] - \frac{1}{2}(\sum_{i,j=1}^{n} \beta_i \beta_j y_i y_j \mathbf{x}_i^T \mathbf{x}_j + \sum_{i=1}^{n} \beta_i^2/\gamma) \qquad (20)
\end{aligned}
$$

$$
\begin{aligned}
\mathbf{I}_2 &= L(\boldsymbol{\alpha}^0) - L(\boldsymbol{\alpha}^0 - \boldsymbol{\delta}) \\
&= \sum_{i=1}^{n} \alpha_i^0 - \frac{1}{2} \sum_{i,j=1}^{n} \alpha_i^0 \alpha_j^0 y_i y_j \mathbf{x}_i^T \mathbf{x}_j - \frac{1}{2\gamma} \sum_{i=1}^{n} (\alpha_i^0)^2 - \sum_{i=1}^{n} (\alpha_i^0 - \delta_i) \\
&\quad + \frac{1}{2} \sum_{i,j=1}^{n} (\alpha_i^0 - \delta_i)(\alpha_j^0 - \delta_j) y_i y_j \mathbf{x}_i^T \mathbf{x}_j + \frac{1}{2\gamma} \sum_{i=1}^{n} (\alpha_i^0 - \delta_i)^2 \\
&= \sum_{i=1}^{n} \delta_i - \sum_{i,j=1}^{n} \alpha_i^0 \delta_j y_i y_j \mathbf{x}_i^T \mathbf{x}_j + \frac{1}{2} \sum_{i,j=1}^{n} \delta_i \delta_j y_i y_j \mathbf{x}_i^T \mathbf{x}_j - \sum_{i=1}^{n} \alpha_i^0 \delta_i/\gamma + \frac{1}{2} \sum_{i=1}^{n} \delta_i^2/\gamma \\
&= \sum_{j=1}^{n} \delta_j (1 - y_j \sum_{i=1}^{n} \alpha_i^0 y_i \mathbf{x}_i^T \mathbf{x}_j - \alpha_j^0/\gamma) + \frac{1}{2}(\sum_{i,j=1}^{n} \delta_i \delta_j y_i y_j \mathbf{x}_i^T \mathbf{x}_j + \sum_{i=1}^{n} \delta_i^2/\gamma) \\
&= \sum_{j=1}^{n} \delta_j (1 - y_j \mathbf{w}_0^T \mathbf{x}_j - \alpha_j^0/\gamma) + \frac{1}{2}(\sum_{i,j=1}^{n} \delta_i \delta_j y_i y_j \mathbf{x}_i^T \mathbf{x}_j + \sum_{i=1}^{n} \delta_i^2/\gamma) \qquad (21)
\end{aligned}
$$

Since $\sum_{i=1}^{n} \delta_i y_i = 0$

$$
\begin{aligned}
&\sum_{j=1}^{n} \delta_j (1 - y_j \mathbf{w}_0^T \mathbf{x}_j - \alpha_j^0/\gamma) + \frac{1}{2}(\sum_{i,j=1}^{n} \delta_i \delta_j y_i y_j \mathbf{x}_i^T \mathbf{x}_j + \sum_{i=1}^{n} \delta_i^2/\gamma) \\
&= \sum_{j=1}^{n} \delta_j [1 - y_j(\mathbf{w}_0^T \mathbf{x}_j + b_0) - \alpha_j^0/\gamma] + \frac{1}{2}(\sum_{i,j=1}^{n} \delta_i \delta_j y_i y_j \mathbf{x}_i^T \mathbf{x}_j + \sum_{i=1}^{n} \delta_i^2/\gamma) \\
&= \frac{1}{2}(\sum_{i,j=1}^{n} \delta_i \delta_j y_i y_j \mathbf{x}_i^T \mathbf{x}_j + \sum_{i=1}^{n} \delta_i^2/\gamma) \qquad (22)
\end{aligned}
$$

Let $\beta_i = \delta_i = \alpha_i^0 - \alpha_i^p \;\; \forall i$, then

$$
\begin{aligned}
\mathbf{I}_1 &= \mathbf{I}_2 \\
&= L(\boldsymbol{\alpha}^0) - L(\boldsymbol{\alpha}^p) \\
&= \beta_p [1 - y_p(\mathbf{w}_p^T \mathbf{x}_p + b_p)] - \frac{1}{2}(\sum_{i,j=1}^{n} \beta_i \beta_j y_i y_j \mathbf{x}_i^T \mathbf{x}_j + \sum_{i=1}^{n} \beta_i^2/\gamma) \\
&= \frac{1}{2}(\sum_{i,j=1}^{n} \delta_i \delta_j y_i y_j \mathbf{x}_i^T \mathbf{x}_j + \sum_{i=1}^{n} \delta_i^2/\gamma) \qquad (23)
\end{aligned}
$$

$$(\alpha_p^0 - \alpha_p^p)[1 - y_p(\mathbf{w}_p^T \mathbf{x}_p + b_p)]$$

$$
\begin{aligned}
&= \alpha_p^0[1 - y_p(\mathbf{w}_p^T\mathbf{x}_p + b_p)] \\
&= \frac{1}{2}(\sum_{i,j=1}^{n} \delta_i\delta_j y_i y_j \mathbf{x}_i^T\mathbf{x}_j + \sum_{i=1}^{n}\delta_i^2/\gamma + \sum_{i,j=1}^{n} \beta_i\beta_j y_i y_j \mathbf{x}_i^T\mathbf{x}_j + \sum_{i=1}^{n}\beta_i^2/\gamma) \\
&= \sum_{i,j=1}^{n} \delta_i\delta_j y_i y_j \mathbf{x}_i^T\mathbf{x}_j + \sum_{i=1}^{n}\delta_i^2/\gamma \\
&= 2[L(\boldsymbol{\alpha}^0) - L(\boldsymbol{\alpha}^p)] \\
&= \sum_{i,j=1}^{n} (\alpha_i^0 - \alpha_i^p)(\alpha_j^0 - \alpha_j^p) y_i y_j \mathbf{x}_i^T\mathbf{x}_j + \sum_{i=1}^{n}(\alpha_i^0 - \alpha_i^p)^2/\gamma \qquad (24)
\end{aligned}
$$

In the leave-one-out procedure, we define the set T_p as a constrained linear combination of all the n-1 training samples $\{\mathbf{x}_i\}_i \neq p$:

$$
T_p = \{ \sum_{i=1, i\neq p}^{n} \lambda_i \mathbf{x}_i : \quad \sum_{i=1, i\neq p}^{n} \lambda_i = 1\} \qquad (25)
$$

We also define a quantity S_p as the square distance between $\mathbf{x}_p$ and this set:

$$
S_p = d^2(\mathbf{x}_p, T_p) = \min_{z\in T_p}(\|\mathbf{x}_p - \mathbf{z}\|)^2 \qquad (26)
$$

By setting $\lambda_p = -1$, we can re-write S_p as:

$$
S_p = \min\{(\| \sum_{i=1}^{n} \lambda_i \mathbf{x}_i\|)^2 : \quad \lambda_p = -1, \sum_{i=1}^{n}\lambda_i = 0\} \qquad (27)
$$

Finally, we define

$$
\min\{\boldsymbol{\lambda}^T(\mathbf{K} + \gamma^{-1}\mathbf{I})\boldsymbol{\lambda} : \quad \lambda_p = -1, \sum_{i=1}^{n}\lambda_i = 0\} = \boldsymbol{\lambda}^{'T}(\mathbf{K} + \gamma^{-1}\mathbf{I})\boldsymbol{\lambda}^{'} \qquad (28)
$$

Based on all these definitions, it is easy to show that:

$$
\begin{aligned}
&(\alpha_p^0 - \alpha_p^p)[1 - y_p(\mathbf{w}_p^T\mathbf{x}_p + b_p)] \\
&= \sum_{i,j=1}^{n} (\alpha_i^0 - \alpha_i^p)(\alpha_j^0 - \alpha_j^p) y_i y_j \mathbf{x}_i^T\mathbf{x}_j + \sum_{i=1}^{n}(\alpha_i^0 - \alpha_i^p)^2/\gamma \\
&= (\alpha_p^0)^2\boldsymbol{\lambda}^{*T}(\mathbf{K} + \gamma^{-1}\mathbf{I})\boldsymbol{\lambda}^{*} \\
&= 2[L(\boldsymbol{\alpha}^0) - L(\boldsymbol{\alpha}^p)] \qquad (29)
\end{aligned}
$$

where $\boldsymbol{\lambda}^*$=$[\lambda_1^*, \lambda_2^*, \ldots, \lambda_n^*]^T$ and $\lambda_i^* = \frac{y_p y_i(\alpha_i^p - \alpha_i^0)}{\alpha_p^0}$

Let $\delta_i = -\alpha_p^0 y_p y_i \lambda_i^{'}$, we have:

$$
2[L(\boldsymbol{\alpha}^0) - L(\boldsymbol{\alpha}^0 - \boldsymbol{\delta})] = (\alpha_p^0)^2\boldsymbol{\lambda}^{'T}(\mathbf{K} + \gamma^{-1}\mathbf{I})\boldsymbol{\lambda}^{'} \qquad (30)
$$

Since $L(\boldsymbol{\alpha}^0) - L(\boldsymbol{\alpha}^p) \leq L(\boldsymbol{\alpha}^0) - L(\boldsymbol{\alpha}^0 - \boldsymbol{\delta})$, then:

$$
(\alpha_p^0)^2\boldsymbol{\lambda}^{*T}(\mathbf{K} + \gamma^{-1}\mathbf{I})\boldsymbol{\lambda}^{*} \leq (\alpha_p^0)^2\boldsymbol{\lambda}^{'T}(\mathbf{K} + \gamma^{-1}\mathbf{I})\boldsymbol{\lambda}^{'}
$$

But accroding to the definitions of $\boldsymbol{\lambda}^*$ and $\boldsymbol{\lambda}^{'}$

$$(\alpha_p^0)^2\boldsymbol{\lambda}^{*T}(\mathbf{K}+\gamma^{-1}\mathbf{I})\boldsymbol{\lambda}^* \geq (\alpha_p^0)^2\boldsymbol{\lambda}^{'T}(\mathbf{K}+\gamma^{-1}\mathbf{I})\boldsymbol{\lambda}^{'}$$

Therefore

$$(\alpha_p^0)^2\boldsymbol{\lambda}^{*T}(\mathbf{K}+\gamma^{-1}\mathbf{I})\boldsymbol{\lambda}^* = (\alpha_p^0)^2\boldsymbol{\lambda}^{'T}(\mathbf{K}+\gamma^{-1}\mathbf{I})\boldsymbol{\lambda}^{'} = \alpha_p^0[1-y_p(\mathbf{w}_p^T\mathbf{x}_p+b_p)]$$

which gives

$$1-y_p(\mathbf{w}_p^T\mathbf{x}_p+b_p) = 1-y_pf^p(\mathbf{x}_p) = \min_{\boldsymbol{\lambda}}\alpha_p^0\boldsymbol{\lambda}^T(\mathbf{K}+\gamma^{-1}\mathbf{I})\boldsymbol{\lambda}$$

Hence Lemma 2 is proved.

Proof of Lemma 1: Since the matrix $\mathbf{K}$+$\gamma^{-1}\mathbf{I}$ is semmetric and positive definite, the minimization problem involved in Eq. (11) can be modified as:

$$\begin{aligned}
&\min_{\boldsymbol{\lambda}}\boldsymbol{\lambda}^T(\mathbf{K}+\gamma^{-1}\mathbf{I})\boldsymbol{\lambda}\\
&=\min_{\lambda_i}(\lambda_p\lambda_p\bar{\mathbf{K}}_{pp}+2\lambda_p\sum_{i=1,i\neq p}^{n}\lambda_i\bar{\mathbf{K}}_{ip}+\sum_{i,j=1,i,j\neq p}^{n}\lambda_i\lambda_j\bar{\mathbf{K}}_{ij})\\
&=\min_{\lambda_i}(\bar{\mathbf{K}}_{pp}-2\sum_{i=1,i\neq p}^{n}\lambda_i\bar{\mathbf{K}}_{ip}+\sum_{i,j=1,i,j\neq p}^{n}\lambda_i\lambda_j\bar{\mathbf{K}}_{ij}) \qquad (31)
\end{aligned}$$

$$s.t. \quad \sum_{i=1,i\neq p}^{n}\lambda_i=1 \qquad (32)$$

where $\bar{\mathbf{K}}$=$\mathbf{K}$+$\gamma^{-1}\mathbf{I}$, and $(\bar{\mathbf{K}})_{ij}$ denotes the ith elements of the jth column of $\bar{\mathbf{K}}$.

By introducing a Lagrangian multiplier μ, we can further modify Eq. (31) as:

$$\begin{aligned}
&\min_{\lambda_i}(\bar{\mathbf{K}}_{pp}-2\sum_{i=1,i\neq p}^{n}\lambda_i\bar{\mathbf{K}}_{ip}+\sum_{i,j=1,i,j\neq p}^{n}\lambda_i\lambda_j\bar{\mathbf{K}}_{ij})\\
&=\min_{\lambda_i}\max_{\mu}[(\bar{\mathbf{K}}_{pp}-2\sum_{i=1,i\neq p}^{n}\lambda_i\bar{\mathbf{K}}_{ip}+\sum_{i,j=1,i,j\neq p}^{n}\lambda_i\lambda_j\bar{\mathbf{K}}_{ij})+2\mu(\sum_{i=1,i\neq p}^{n}\lambda_i-1)]\\
&=\min_{\lambda_i}\max_{\mu}(\bar{\mathbf{K}}_{pp}-2\mathbf{H}_{/pp}\bar{\boldsymbol{\lambda}}+\bar{\boldsymbol{\lambda}}^T\mathbf{H}_{/p}\bar{\boldsymbol{\lambda}})
\end{aligned}$$

where $\bar{\boldsymbol{\lambda}}=[\lambda_1,\lambda_2,\ldots,\lambda_{p-1},\lambda_{p+1},\ldots,\lambda n,\mu]^T$, $\mathbf{H}$=$\begin{bmatrix}\mathbf{K}+\gamma^{-1}\mathbf{I} & \vec{\mathbf{1}}\\ \vec{\mathbf{1}}^T & 0\end{bmatrix}$, $\mathbf{H}_{/pp}$ is the pth column of $\mathbf{H}$ with the pth entry removed and $\mathbf{H}_{/p}$ is the submatrix of $\mathbf{H}$ with row and column p removed.

From the fact that the optimal value of $\bar{\boldsymbol{\lambda}}$ is $(\mathbf{H}_{/p})^{-1}\mathbf{H}_{/pp}$, it follows

$$\min_{\lambda_i}\max_{\mu}(\bar{\mathbf{K}}_{pp}-2\mathbf{H}_{/pp}\bar{\boldsymbol{\lambda}}+\bar{\boldsymbol{\lambda}}^T\mathbf{H}_{/p}\bar{\boldsymbol{\lambda}})=\bar{\mathbf{K}}_{pp}-\mathbf{H}_{/pp}^T(\mathbf{H}_{/p})^{-1}\mathbf{H}_{/pp}$$

Since for a block matrix, there is:

$$\begin{bmatrix} \mathbf{A}_{11} & \mathbf{A}_{12} \\ \mathbf{A}_{21} & \mathbf{A}_{22} \end{bmatrix}^{-1} = \begin{bmatrix} (\mathbf{A}_{11} - \mathbf{A}_{12}\mathbf{A}_{22}^{-1}\mathbf{A}_{21})^{-1} & -\mathbf{A}_{11}^{-1}\mathbf{A}_{12}\mathbf{F}_{22}^{-1} \\ -\mathbf{F}_{22}^{-1}\mathbf{A}_{21}\mathbf{A}_{11}^{-1} & \mathbf{F}_{22}^{-1} \end{bmatrix}$$

by resorting the matrix $\mathbf{H}$ (change the position of the rows and columns in the matrix), we can get:

$$(\mathbf{H}^{-1})_{pp} = (\bar{\mathbf{K}}_{pp} - \mathbf{H}_{/pp}^T(\mathbf{H}_{/p})^{-1}\mathbf{H}_{/pp})^{-1} \tag{33}$$

Therefore

$$\begin{aligned} 1 - y_p f^p(\mathbf{x}_p) &= \alpha_p^0 \min_{\boldsymbol{\lambda}} \boldsymbol{\lambda}^T(\mathbf{K} + \gamma^{-1}\mathbf{I})\boldsymbol{\lambda} \\ &= \alpha_p^0 \min_{\lambda_i} \max_{\mu} (\bar{\mathbf{K}}_{pp} - 2\mathbf{H}_{/pp}\bar{\boldsymbol{\lambda}} + \bar{\boldsymbol{\lambda}}^T\mathbf{H}_{/p}\bar{\boldsymbol{\lambda}}) \\ &= \frac{\alpha_p^0}{(\mathbf{H}^{-1})_{pp}} \end{aligned}$$

and Lemma 1 is proved

4 The partial derivative of LLOOC

Let $\bar{\boldsymbol{\Omega}}$=$\boldsymbol{\Omega} + \gamma^{-1}\mathbf{I}$, and $\mathbf{M}$=$\begin{bmatrix} \boldsymbol{\Omega} + \gamma^{-1}\mathbf{I} & \mathbf{Y} \\ \mathbf{Y}^T & 0 \end{bmatrix}$, we can get

$$\boldsymbol{\alpha} = \bar{\boldsymbol{\Omega}}^{-1}(\vec{\mathbf{1}} - \mathbf{Y}b) \tag{34}$$

$$b = \frac{\mathbf{Y}^T\bar{\boldsymbol{\Omega}}^{-1}\vec{\mathbf{1}}}{\mathbf{Y}^T\bar{\boldsymbol{\Omega}}^{-1}\mathbf{Y}} \tag{35}$$

which gives:

$$\boldsymbol{\alpha} = \bar{\boldsymbol{\Omega}}^{-1}\vec{\mathbf{1}} - \frac{\bar{\boldsymbol{\Omega}}^{-1}\mathbf{Y}\mathbf{Y}^T\bar{\boldsymbol{\Omega}}^{-1}\vec{\mathbf{1}}}{\mathbf{Y}^T\bar{\boldsymbol{\Omega}}^{-1}\mathbf{Y}} \tag{36}$$

Let $g_i = 1 - \alpha_i/(\mathbf{H}^{-1})_{ii}$ be the LOO test result of the LS-SVM on sample $\mathbf{x}_i$, the LLOOC measure is:

$$\mathbf{LLOOC} = \frac{1}{n}\sum_{i=1}^{n} \frac{1}{1 + exp(g_i)}$$

Then the partial derivative of **LLOOC** with respect to any parameter t is given by:

$$\begin{aligned} &\frac{\partial \mathbf{LLOOC}}{\partial t} = -\frac{1}{n}\sum_{i=1}^{n} \frac{exp(g_i)}{[1 + exp(g_i)]^2} \cdot \frac{\partial g_i}{\partial t} \\ &= -\frac{1}{n}\sum_{i=1}^{n} \frac{exp(g_i)}{[1 + exp(g_i)]^2} \cdot [\frac{\partial g_i}{\partial t}|_{\boldsymbol{\alpha},\mathbf{H}_{fixed}} + \frac{\partial g_i}{\partial \alpha_i} \cdot \frac{\partial \alpha_i}{\partial t} + \frac{\partial g_i}{\partial (\mathbf{H}^{-1})_{ii}} \cdot \frac{\partial (\mathbf{H}^{-1})_{ii}}{\partial t}] \\ &= \frac{1}{n}\sum_{i=1}^{n} \frac{exp(g_i)}{[1 + exp(g_i)]^2} \cdot [\frac{1}{(\mathbf{H}^{-1})_{ii}} \cdot \frac{\partial \alpha_i}{\partial t} - \frac{\alpha_i}{(\mathbf{H}^{-1})_{ii}^2} \cdot \frac{\partial (\mathbf{H}^{-1})_{ii}}{\partial t}] \end{aligned} \tag{37}$$

Because $\frac{\partial(\mathbf{H}^{-1})}{\partial t} = -\mathbf{H}^{-1}\frac{\partial \mathbf{H}}{\partial t}\mathbf{H}^{-1}$, Eq. (37) can be re-written as

$$\frac{\partial \mathbf{LLOOC}}{\partial t} = \frac{1}{n(\mathbf{H}^{-1})^2_{ii}} \sum_{i=1}^{n} \frac{exp(g_i)}{[1+exp(g_i)]^2}[(\mathbf{H}^{-1})_{ii} \cdot \frac{\partial \alpha_i}{\partial t} - \alpha_i(\mathbf{H}^{-1}\frac{\partial \mathbf{H}}{\partial t}\mathbf{H}^{-1})_{ii}]$$

Further, because $\begin{bmatrix} \boldsymbol{\alpha} \\ b \end{bmatrix} = \mathbf{M}^{-1}\begin{bmatrix} \vec{\mathbf{1}} \\ 0 \end{bmatrix}$, we have:

$$\frac{\partial(\boldsymbol{\alpha}, b)}{\partial t} = -\mathbf{M}^{-1}\frac{\partial \mathbf{M}}{\partial t}\mathbf{M}^{-1}\begin{bmatrix} \vec{\mathbf{1}} \\ 0 \end{bmatrix} \tag{38}$$

$$\frac{\partial \alpha_i}{\partial t} = (-\mathbf{M}^{-1}\begin{bmatrix} \frac{\partial \bar{\mathbf{\Omega}}}{\partial t} & \mathbf{0} \\ \mathbf{0} & 0 \end{bmatrix}\mathbf{M}^{-1}\begin{bmatrix} \vec{\mathbf{1}} \\ 0 \end{bmatrix})_i \tag{39}$$

where $(\mathbf{x})_i$ denotes the ith elements of vector $\mathbf{x}$

By substituting $\frac{\partial \alpha_i}{\partial t}$ and $\frac{\partial(\mathbf{H}^{-1})}{\partial t}$ into Eq. (37), we can finally get:

$$\frac{\partial \mathbf{LLOOC}}{\partial t} = \frac{1}{n(\mathbf{H}^{-1})^2_{ii}} \sum_{i=1}^{n} \frac{exp(1-\alpha_i/(\mathbf{H}^{-1})_{ii})}{[1+exp(1-\alpha_i/(\mathbf{H}^{-1})_{ii})]^2}$$
$$[\alpha_i(\mathbf{H}^{-1}\begin{bmatrix} \frac{\partial \mathbf{K}}{\partial v_k} & \mathbf{0} \\ \mathbf{0} & 0 \end{bmatrix}\mathbf{H}^{-1})_{ii} - \mathbf{H}^{-1}_{ii}(\mathbf{M}^{-1}\begin{bmatrix} \frac{\partial \mathbf{\Omega}}{\partial t} & \mathbf{0} \\ \mathbf{0} & 0 \end{bmatrix}\mathbf{M}^{-1}\begin{bmatrix} \vec{\mathbf{1}} \\ 0 \end{bmatrix})_i]$$
